# ets1 associates with KMT5A to participate in high glucose-mediated EndMT via upregulation of PFN2 expression in diabetic nephropathy

**DOI:** 10.1186/s10020-021-00339-7

**Published:** 2021-07-08

**Authors:** Lihong Lu, Ziwen Zhong, Jiahui Gu, Ke Nan, Minmin Zhu, Changhong Miao

**Affiliations:** 1 Department of Anesthesiology, Department of Oncology, Fudan University Shanghai Cancer Center, Shanghai Medical College, Fudan University, Shanghai, 200032 China; 2 Department of Anesthesiology, Zhongshan Hospital, Fudan University, Shanghai, 200032 China; 3 Department of Anesthesiology, Affiliated Hospital of North Sichuan Medical College, Nanchong, China

**Keywords:** KMT5A, ets1, PFN2, EndMT, Diabetic nephropathy

## Abstract

**Background:**

Diabetic nephropathy (DN) is currently the leading cause of end-stage renal disease globally. The endothelial-to-mesenchymal transition (EndMT) of glomerular endothelial cells has been reported to play a crucial role in DN. As a specific form of epithelial-to-mesenchymal transition, EndMT and epithelial-to-mesenchymal transition may exhibit mutual modulators. Profilin 2 (PFN2) has been reported to participate in epithelial-to-mesenchymal transition. Moreover, ETS proto-oncogene 1 (ets1) and lysine methyltransferase 5A (KMT5A) have been reported to contribute to high glucose-mediated endothelial injury and epithelial-to-mesenchymal transition. In this study, we hypothesize ets1 associates with KMT5A to modulate PFN2 transcription, thus participating in high glucose-mediated EndMT in glomerular endothelial cells.

**Methods:**

Immunohistochemistry (IHC) was performed to detect protein levels in the kidney tissues and/or aorta tissues of human subjects and rats. Western blot, qPCR and immunofluorescence were performed using human umbilical vein endothelial cells (HUVECs). Chromatin immunoprecipitation (ChIP) assays and dual luciferase assays were performed to assess transcriptional activity. The difference between the groups was compared by two-tailed unpaired t-tests or one-way ANOVAs.

**Results:**

Our data indicated that vimentin, αSMA, S100A4 and PFN2 levels were increased, and CD31 levels were reduced in glomerular endothelial cells of DN patients and rats. Our cell experiments showed that high glucose induced EndMT by augmenting PFN2 expression in HUVECs. Moreover, high glucose increased ets1 expression. si-ets1 suppressed high glucose-induced PFN2 levels and EndMT. ets1 overexpression-mediated EndMT was reversed by si-PFN2. Furthermore, ets1 was determined to associate with KMT5A. High glucose attenuated KMT5A levels and histone H4 lysine 20 methylation (H4K20me1), one of the downstream targets of KMT5A. KMT5A upregulation suppressed high glucose-induced PFN2 levels and EndMT. sh-KMT5A-mediated EndMT was counteracted by si-PFN2. Furthermore, H4K20me1 and ets1 occupied the PFN2 promoter region. sh-KMT5A cooperated with ets1 overexpression to activate PFN2 promoter activity. Our in vivo study demonstrated that KMT5A was reduced, while ets1 was augmented, in glomerular endothelial cells of DN patients and rats.

**Conclusions:**

The present study indicated that ets1 cooperated with KMT5A to transcribe PFN2, thus contributing to hyperglycemia-induced EndMT in the glomerular endothelial cells of DN patients and rats.

*Trial registration* ChiCTR, ChiCTR2000029425. 2020/1/31, http://www.chictr.org.cn/showproj.aspx?proj=48548

**Supplementary Information:**

The online version contains supplementary material available at 10.1186/s10020-021-00339-7.

## Background

Diabetic nephropathy (DN) has become one of the primary causes of mortality in diabetic patients and may result in end-stage renal disease (Packham et al. 2012; Tomino and Gohda 2015). DN is characterized by a gradual elevation of urine albumin, a rise in blood pressure, and a decline of the glomerular filtration rate (Boer et al. 2011; Ng et al. 2016). However, current treatment strategies can only delay and cannot prevent DN progression. Once DN develops to end-stage renal disease, the cost of treatment and cardiovascular-related mortality both increase (Alves and Lewis 2010; Xue et al. 2017). Therefore, the search for the potential mechanisms underlying DN is urgent. The endothelial-to-mesenchymal transition (EndMT) of glomerular endothelial cells has been reported to play a crucial role in the occurrence and progression of DN (Li et al. 2010; Kanasaki et al. 2014).

EndMT is an intricate cellular differentiation process whereby endothelial cells detach and migrate away from the endothelium, lose endothelial properties and acquire mesenchymal features. EndMT is defined as endothelial cell loss of the endothelial phenotype, including CD31, and acquisition of mesenchymal features, including vimentin (Liang et al. 2016). As EndMT is a specific form of epithelial-to-mesenchymal transition, it is possible that epithelial-to-mesenchymal transition modulators also play an important role in the modulation of EndMT (Saito 2013).

Profilins are well known as actin-binding proteins (Ding et al. 2012). Previous studies have indicated that profilin2 (PFN2) participates in epithelial-to-mesenchymal transition in cancer cells (Tang et al. 2015; Zhang et al. 2018). However, whether PFN2 participates in hyperglycemia-mediated EndMT in endothelial cells has not been reported.

ets1, a member of the ETS family of transcription factors, plays roles in kidney development and glomerular integrity (Razzaque et al. 2001). Moreover, ets1 serves as a crucial transcription factor in the progression of DN (Geng et al. 2019; Liu et al. 2011). ets1 suppression was reported to attenuate angiotensin II-induced cardiac fibrosis via the inhibition of EndMT (Xu et al. 2019). EST1 may be a potential drug target for the treatment of diabetic nephropathy.

Lysine methyltransferase 5A (KMT5A) is the only known nucleosome-specific methyltransferase that modulates histone H4 lysine 20 through methylation (H4K20me1) (Beck et al. 2012). Our previous studies have shown that KMT5A downregulation participates in high glucose-mediated vascular endothelial injury (Chen et al. 2018, 2020a, b; Qi et al. 2020; Wang et al. 2020; Shen et al. 2020). Moreover, KMT5A was reported to mediate epithelial-to-mesenchymal transition (Hou et al. 2016). However, whether KMT5A is involved in high glucose-induced EndMT is still not well known. In the present study, we hypothesize that ets1 may associate with KMT5A to modulate PFN2 expression, thus performing a vital role in high glucose-mediated EndMT in glomerular endothelium.

## Methods

### Subjects

Twenty diagnosed DN patients (type II diabetes) and twenty control participants (renal cancer patients with normal renal function) were consecutively recruited. This research abided by the Declaration of Helsinki and was authorized by the Ethics Committee of Huzhou Central Hospital. The license number of the present study is 20191209-01. Written informed consent was acquired from all the participants recruited. The Chronic Kidney Disease Epidemiology Collaboration equation (CKD-EPI) was used to calculate the glomerular filtration of human subjects. DN patients’ staging protocol was defined according to the National Kidney Foundation’s Kidney Disease Outcomes Quality Initiative classification scheme.

### Rat model of DN

Male Sprague–Dawley rats weighing 180–200 g were selected for the present study. The animals were purchased from Shanghai SLAC Laboratories. This study abided by the Guide for the Care and Use of Laboratory Animals of Shanghai Medical College at Fudan University. Animals were housed in the care facilities as described previously (Wang et al. 2020; Shen et al. 2020). Twenty rats were randomly assigned to the control group (con, n = 10) and the DN group (DN, n = 10). In the control group, the animals received a single intraperitoneal injection of citrate buffer (0.1 M, pH 4.5). In the DN group, after being raised with a high sugar-fat diet for 2 weeks, the animals were intraperitoneally injected once with streptozotocin (STZ, 50 mg/kg). Then, the animals were sent back to the care facilities for 6 weeks. Three days later, hyperglycemia in the DN group animals was confirmed by monitoring tail vein blood samples.

### Collection of rat blood samples and urine specimens

One day before euthanasia, 24-h urine was collected, and albumin levels were detected after urine collection. Euthanasia of all the animals was carried out by intraperitoneal injection of thiopental sodium (40 mg/kg). Animal blood samples were gathered by cardiac puncture. After collection, the plasma and urine specimens were preserved at −80 °C until detection. Serum creatinine levels were detected with the use of a creatinine assay kit (Jiancheng Bio, Nanjing, China). Serum urea nitrogen (BUN) levels were detected with the use of a BUN assay kit (Jiancheng Bio, Nanjing, China). Twenty-four-hour urinary albumin levels were detected with the use of an enhanced BCA protein assay kit (Beyotime, Shanghai, China).

### Hematoxylin–eosin (HE) staining, Masson staining and immunohistochemistry (IHC)

The tissues of human subjects and rats were wrapped in paraffin and then processed for HE, Masson staining and IHC. HE was used to detect histopathological abnormalities. Masson staining was used to determine the severity of fibrosis according to the kit instructions (Solarbrio, Beijing, China). The paraffin sections were incubated with antibodies against KMT5A (Proteintech, Wuhan, China), ets1 (Proteintech, Wuhan, China), PFN2 (Proteintech, Wuhan, China), vimentin (Proteintech, Wuhan, China), S100A4 (Proteintech, Wuhan, China), αSMA (Proteintech, Wuhan, China), and CD31 (Proteintech, Wuhan, China) at 4 °C for 12 h.

### Cell culture and reagents

Human umbilical vein endothelial cells (HUVECs) were purchased from American Type Culture Collection (ATCC; Manassas, USA). The cells were incubated in Dulbecco’s modified Eagle medium (DMEM; HyClone Laboratories, USA) with 5 mM glucose as a control group (con). In the high glucose (HG) treatment group, cells were cultured in DMEM (HyClone Laboratories, USA) with 25 mM glucose. Glucose (5 mM) plus mannitol (20 mM) was used as an osmotic control.

### Western blot analysis

Cell protein extracts were obtained using cell lysis buffer (Jiancheng Bio, Nanjing, China). Different groups of HUVECs with equal amounts of proteins (50 μg) were separated by 8–12% SDS-PAGE and transferred to PVDF membranes (Millipore, Billerica, USA). The membranes were incubated in a 5% skimmed milk solution at room temperature for 1 h. Then, all membranes were incubated with the corresponding primary antibodies at 4 °C for 12 h. The primary antibodies used in the present study were as follows: monoclonal antibodies against β-actin (Proteintech, Wuhan, China), KMT5A (Proteintech, Wuhan, China), H4K20me1 (Abcam, Cambridge, UK), ets1 (Proteintech, Wuhan, China), PFN2 (Proteintech, Wuhan, China), αSMA (Proteintech, Wuhan, China), S100A4 (Proteintech, Wuhan, China), CD31 (Proteintech, Wuhan, China) and vimentin (Proteintech, Wuhan, China). Then, an HRP-conjugated secondary antibody was employed. The protein signal was determined by an ECL system (Shanghai Epizyme Biomedical Technology Co. Ltd., Shanghai, China).

### RNA extraction and quantitative real-time PCR (qPCR)

Cell and tissue RNA was extracted by using TRIzol reagent (Invitrogen, Grand Island, NY, USA). Complementary DNA (cDNA) was synthesized with the use of PrimeScript RT reagent (TaKaRa). Then, we performed qPCR via Hieff UNICON® qPCR TaqMan Probe Master Mix (Yeasen, Shanghai, China) on an ABI7500 Real-Time PCR system (Applied Biosystems). The primers employed are listed in Additional file 1: Table S1.

### Bioinformatics analysis

In the present study, inbio-discover website was used to explore the potential proteins that interact with ets1. Then, these proteins were retrieved and loaded into R.studio for gene ontology (GO) mapping and annotation (p < 0.01 was the cut-off criterion).

### Coimmunoprecipitation (CoIP)

Cell protein extracts were obtained with cell lysis buffer containing PMSF (Jiancheng Bio, Nanjing, China). Then, 30 μl of the cell protein extracts were employed as input. For endogenous IP, cell protein extracts were incubated with specific primary antibodies and 50 μl protein A/G Dynabeads (Thermo Fisher, USA) at 4 °C for 12 h. Then, 8 μl of input, IgG and IP extracts were processed for Western blotting.

### Immunofluorescence (IF) staining

HUVECs were plated onto glass slides with the corresponding treatment. After fixation with 4% paraformaldehyde, HUVECs were permeabilized with 0.3% Triton X-100 for 5 min. Then, the cells were blocked with 1% bovine serum albumin at room temperature for 1 h. Then, the cells were incubated with specific primary antibodies at 4 °C for 12 h. 4,6-Diamidino-2-phenylindole (DAPI) was employed to stain the nucleus.

### siRNA, shRNA and KMT5A mutant treatments

HUVECs were transfected with sh-KMT5A, a mutant KMT5A^R295G^ plasmid (Yang et al. 2012), si-ets1 and si-PFN2 using Lipofectamine 3000 (Invitrogen, USA). The sequences of sh-KMT5A (Biotend, Shanghai, China) were shRNA-a, 5′-CAACAGAATCGCAAACTTA-3′, and shRNA-b, 5′-CAACAGAATCGCAAACTTA-3′. The sequences of si-ets1 (Biotend, Shanghai, China) were siRNA-a, 5′-CGCUAUACCUCGGAUUACUdTdT-3′, and siRNA-b, 5′-CCACUAUUAACUCCAAGCAdTdT-3′. The sequences of si-PFN2 (Biotend, Shanghai, China) were siRNA-a, 5′-GGCAGAGCUACGUGGAUAAdTdT-3′, and siRNA-b, 5′-GCGAAGAAAUGCUCAGUGAdTdT-3′.

### Chromatin immunoprecipitation (ChIP) assay

ChIP assays were performed with the use of a Simple ChIP Plus Sonication Chromatin IP Kit (Cell Signaling Technology, MA) as described previously (Wang et al. 2020; Shen et al. 2020). In brief, HUVECs (1 × 10^7^) were fixed with 1% formaldehyde at room temperature for 10 min. Then, the reaction process was terminated by glycine. Chromatin was sheared with the use of a Microson XL ultrasonic cell disruptor XL (Misonix). Then, 10 μl of ultrasonic solution was employed as input. The rest of the ultrasonic solution was cultured with specific antibodies against ets1 (Proteintech, Wuhan, China) and H4K20me1 (Abcam, Cambridge, UK), as well as a negative control IgG at 4 °C for 12 h. After protein G magnetic beads absorbed the immunoprecipitants, the DNA–protein interaction was terminated by incubation at 65 °C for 2 h. After purification, the enriched DNA sequences were determined by PCR. The PFN2 oligonucleotide primers were as follows: forward, 5′-TGGGGGACCCCTAATTCCAT-3′, and reverse, 5’-GGACCCAGGTCTGGGAAAGA-3′.

### Dual-luciferase assay

The activity of the PFN2 promoter was assessed by a Promega Dual-luciferase Assay Kit (Madison, WI, USA) as described previously (Wang et al. 2020). The PFN2 promoter was connected to the pGL3-basic vector, thus establishing the pGL3-PFN2 construct. HUVECs were transfected with the pGL3-PFN2 plasmid. The promoter activity of PFN2 was evaluated with the corresponding treatments.

### Statistical analysis

In the present study, the sample sizes were calculated by evaluation of the effect of hyperglycemia or high glucose on PFN2 mRNA expression, which was detected in our pilot in vivo or in vitro experiments. Statistical significance may be acquired with a sample size of 10 in in vivo experiments and 5 in in vitro experiments.

The results are expressed as the mean ± standard deviation. The difference between the groups was compared by two-tailed unpaired *t*-tests or one-way ANOVAs with GraphPad Prism Version 7.0 (GraphPad Software, San Diego, CA). P < 0.05 was considered significant.

## Results

### Occurrence of EndMT and increase in PFN2 expression in DN patients and rats

**Fig. 1:**
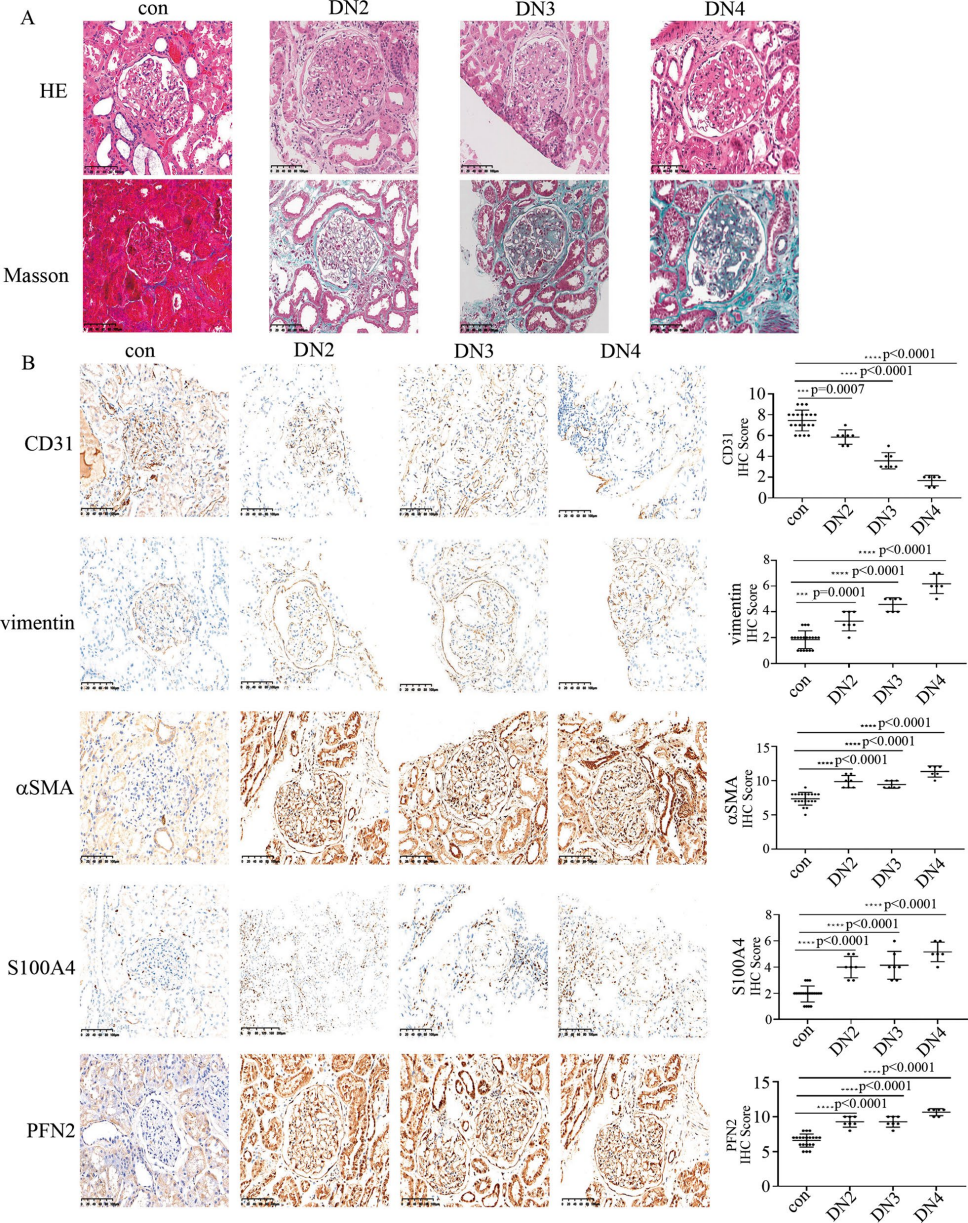
Occurrence of EndMT and increase in PFN2 expression in DN patients. **A** HE staining and Masson staining in renal biopsy specimens of DN patients and control participants. Magnification: ×20. Scale bar: 20 μM. (* p < 0.05, ** p < 0.01, *** p < 0.001, **** p < 0.0001, n = 20 for the control group, n = 7 for the DN2 group, n = 7 for the DN3 group, n = 6 for the DN4 group) **B** Immunostaining of CD31, vimentin, αSMA and S100A4 and PFN2 in renal biopsy specimens of DN patients and control participants. Magnification: ×20. Scale bar: 20 μM. (* p < 0.05, ** p < 0.01, *** p < 0.001, **** p < 0.0001, n = 20 for the control group, n = 7 for the DN2 group, n = 7 for the DN3 group, n = 6 for the DN4 group)

The characteristics of the control and DN participants are shown in Table 1. The DN patients recruited in the present study were in DN stage 2 (DN2), DN stage 3 (DN3) or DN stage 4 (DN4) according to the eGFR. HE staining of the participants is shown in Fig. 1A. Masson staining resulted in more blue staining in the renal biopsy specimens of DN patients, which represented collagen deposition and extensive interstitial fibrosis (Fig. 1A). EndMT in glomerular endothelial cells has been reported to play a crucial role in DN and fibrosis (Li et al. 2010; Kanasaki et al. 2014). Therefore, we detected CD31, vimentin, αSMA and S100A4 in renal biopsy specimens of DN patients and control participants. Compared with control participants, CD31 expression was decreased, while vimentin, αSMA and S100A4 expression was increased in glomerular endothelial cells of DN patients (Fig. 1B). More importantly, our data indicated that vimentin, αSMA, and S100A4 gradually increased, while CD31 gradually decreased in glomeruli when the DN stage progressed (Fig. 1B).

**Table 1:** Characteristics of participants and rats in control (con) and Diabetic Nephropathy (DN) group

Human															
Subjects	Age (Year)	BMI (kg/m2)	SBP (mmHg)		DBP (mmHg)	HbA1C (%)	FBG (mmol/l)		CREA (umol/l)	ALB (g/L)	CCr (ml/min)		24hUPQ (mg)	TP (g/L)	eGFR (ml/min/)
Normal control (n = 20)															
Male: 9/20	58.3 ± 7.3	23.4 ± 3.3	118.9 ± 15.2		69.2 ± 8.1	5.2 ± 0.8	5.2 ± 0.5		58.3 ± 10.5	47.2 ± 5.2	101.9 ± 10.8		95.5 ± 10.2	69.5 ± 7.3	130.1 ± 25.2
DN stage 2 (n = 7)															
Male: 3/7	57.4 ± 4.7	22.5 ± 2.3	133 ± 10.2		75.3 ± 8.3	6.8 ± 1.2	6.4 ± 0.8		100.2 ± 10.3	35.4 ± 3.2	80.9 ± 7.6		1499.4 ± 234.8	55.4 ± 6.1	71.4 ± 6.8
DN stage 3 (n = 7)															
Male: 4/7	60.2 ± 4.4	24.6 ± 2.5	142.8 ± 12.1		78.3 ± 8.2	7.2 ± 2.0	7.2 ± 2.3		155.9 ± 12.4	32.2 ± 2.9	65.5 ± 7.1		4233.4 ± 359.0	50.8 ± 5.0	48.5 ± 6.5
DN stage 4 (n = 6)															
Male: 3/6	59.1 ± 5.9	21 ± 4.3	147.3 ± 6.2		85.1 ± 6.9	8.9 ± 2.5	9.2 ± 3.1		265.8 ± 20.9	27.5 ± 3.6	39.8 ± 4.6		6750.4 ± 675.3	45.7 ± 8.0	23.4 ± 5.1
Rats															
Variables				Con				DM				P-value			
CREA (umol/l)				21.0 ± 2.2				39.7 ± 9.7				< 0.0001			
UREA (mmol/l)				2.3 ± 0.2				6.4 ± 1.1				< 0.0001			
24hUTP (mg)				31.7 ± 4.5				357.9 ± 70.3				< 0.0001			

Data are presented as means ± SD. BMI (Body Mass Index), SBP (systolic blood pressure), DBP (diastolic blood pressure), HbA1c (glycated hemoglobin), FBG (fasting blood glucose), CREA (creatinine), ALB (albumin), CCr (Creatinine Clearance), 24hUPQ (24-h urinary protein quantity), TP (Total Protein), eGFR (Estimated Glomerular Filtration Rate)

PFN2 was reported to be involved in epithelial-to-mesenchymal transition (Tang et al. 2015; Zhang et al. 2018). Next, we detected PFN2 expression and found that PFN2 expression in glomerular endothelial cells was higher in DN patients than in control participants (Fig. 1B). Moreover, PFN2 was found to gradually increase in glomeruli when the DN stage progressed (Fig. 1B). These data indicated that as DN progresses, so does vascular injury.

The characteristics of the control and DN rats are listed in Table 1. Accordingly, HE staining and Masson trichrome staining indicated renal injury and interstitial fibrosis in the glomerular endothelial cells of DN rats (Additional file 2: Figure S1A). Moreover, the levels of PFN2, vimentin, αSMA and S100A4 in the kidney tissues (Additional file 2: Figure S1B) of DN rats increased compared with the levels in the control group. The levels of CD31 in the kidney tissues (Additional file 2: Figure S1B) of DN rats were lower than the levels of CD31 in the kidney tissues of the control group. Furthermore, the protein and mRNA levels of PFN2, vimentin, αSMA and S100A4 in aortic tissues (Additional file 3: Figure S2A, C-F) of DN rats were higher than the protein and mRNA levels of PFN2, vimentin, αSMA and S100A4 in aortic tissues of the control group. The protein and mRNA levels of CD31 in aortic tissues (Additional file 3: Figure S2A, B) of DN rats were lower than the protein and mRNA levels of CD31 in aortic tissues of the control group. The blood glucose evolution of rats in this study is provided in Additional file 3: Figure S2G. These data may indicate that EndMT and PFN2 participate in the occurrence and progression of DN.

### High-glucose treatment induced EndMT via an increase in PFN2 levels in HUVECs

**Fig. 2:**
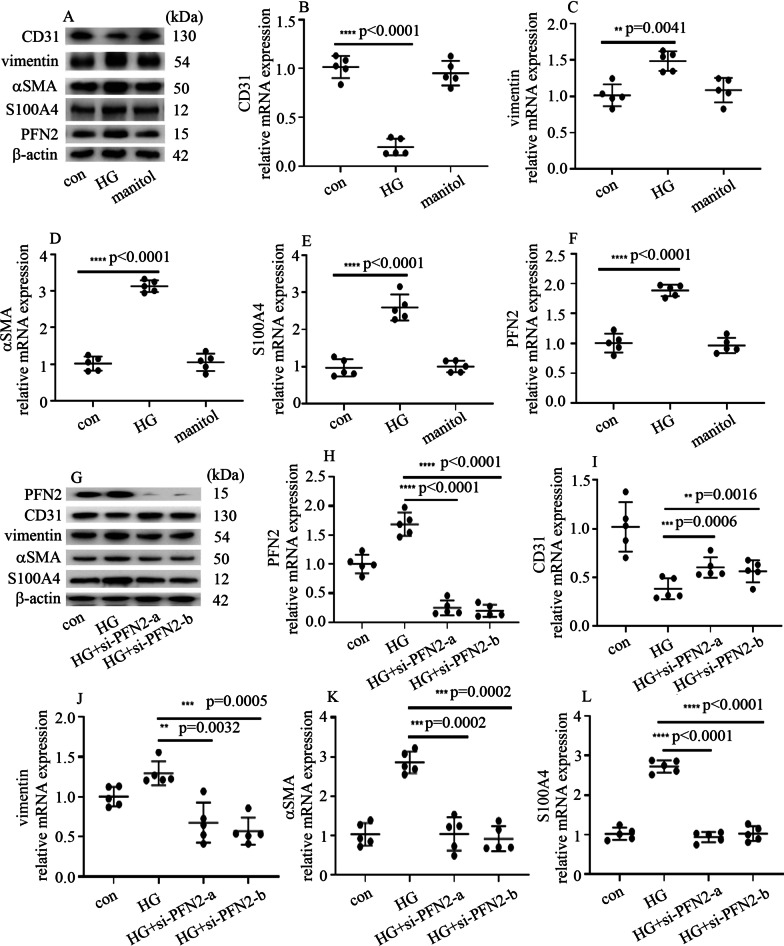
High-glucose treatment mediated EndMT via the upregulation of PFN2 expression in HUVECs. **A** Results from the Western blot analysis of CD31, vimentin, αSMA, S100A4 and PFN2 levels in HUVECs with the corresponding treatment. **B** Compared with the control group, the mRNA expression of CD31 was decreased in hyperglycemic HUVECs. **C** Compared with the control group, the mRNA expression of vimentin was increased in hyperglycemic HUVECs. **D** Compared with the control group, the mRNA expression of αSMA was increased in hyperglycemic HUVECs. **E** Compared with the control group, the mRNA expression of S100A4 was increased in hyperglycemic HUVECs. **F** Compared with the control group, the mRNA expression of PFN2 was increased in hyperglycemic HUVECs. **G** Results from the Western blot analysis of PFN2, CD31, vimentin, αSMA and S100A4 levels in HUVECs with the corresponding treatment. **H** The effects of si-PFN2 were confirmed by qPCR. **I** Compared with high-glucose treatment, si-PFN2 increased CD31 mRNA expression in hyperglycemic HUVECs. **J** Compared with high-glucose treatment, si-PFN2 decreased vimentin mRNA expression in hyperglycemic HUVECs. **K** Compared with high-glucose treatment, si-PFN2 decreased αSMA mRNA expression in hyperglycemic HUVECs. **L** Compared with high-glucose treatment, si-PFN2 decreased S100A4 mRNA expression in hyperglycemic HUVECs. (* p < 0.05, ** p < 0.01, *** p < 0.001, **** p < 0.0001, n = 5/group)

To explore whether PFN2 modulated EndMT in the glomerular endothelial cells of DN patients and rats, HUVECs were employed in the present study. To determine whether a high glucose level participated in the induction of EndMT and PFN2 expression in HUVECs, cells were cultured in DMEM with 5 mM glucose (con, 5 mM, 6 days) or DMEM with 25 mM glucose (HG, 25 mM, 6 days). Our data showed that high-glucose treatment inhibited CD31 expression while increasing vimentin, αSMA and S100A4 expression in HUVECs (Fig. 2A-E). These data indicated that high-glucose treatment mediated EndMT in HUVECs. Mannitol treatment did not affect CD31, vimentin, αSMA or S100A4 expression (Fig. 2A-E). Previous research indicated that PFN2 participates in epithelial-to-mesenchymal transition (Tang et al. 2015; Zhang et al. 2018). Therefore, PFN2 expression was detected in HUVECs. The results indicated that high-glucose treatment augmented PFN2 protein (Fig. 2A) and mRNA expression (Fig. 2F) in HUVECs. To further illustrate whether PFN2 plays an important role in high glucose-mediated EndMT, two independent siRNAs against PFN2 were used in the present study. The effects of si-PFN2 were verified by Western blotting (Fig. 2G) and qPCR (Fig. 2H). Our data demonstrated that si-PFN2 counteracted the high glucose-mediated decrease in CD31 expression and the increase in vimentin, αSMA and S100A4 expression (Fig. 2G, I-L). These data indicated that PFN2 positively modulated EndMT in hyperglycemic HUVECs.

### ets1 participated in high glucose-induced EndMT by augmenting PFN2 expression in HUVECs

**Fig. 3:**
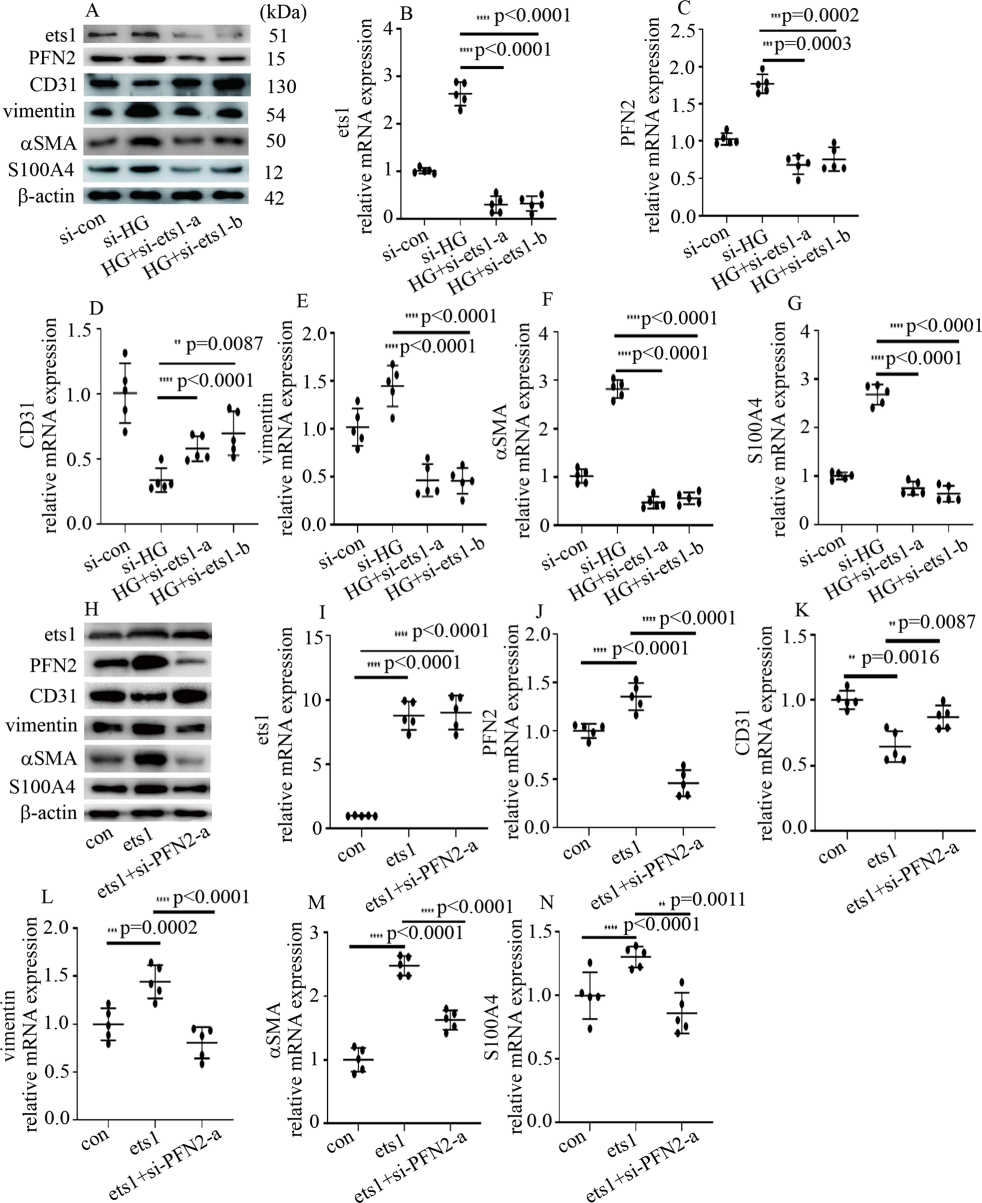
ets1 participated in high glucose-induced EndMT by augmenting PFN2 expression in HUVECs. **A** Results from the Western blot analysis of ets1, PFN2, CD31, vimentin, αSMA and S100A4 in the HUVECs with the corresponding treatment. **B** The effects of si-ets1 were confirmed by qPCR. **C** Compared with high-glucose treatment, si-ets1 decreased PFN2 mRNA expression in hyperglycemic HUVECs. **D** Compared with high-glucose treatment, si-ets1 increased CD31 mRNA expression in hyperglycemic HUVECs. **E** Compared with high-glucose treatment, si-ets1 decreased vimentin mRNA expression in hyperglycemic HUVECs. **F** Compared with high-glucose treatment, si-ets1 decreased αSMA mRNA expression in hyperglycemic HUVECs. **G** Compared with high-glucose treatment, si-ets1 decreased S100A4 mRNA expression in hyperglycemic HUVECs. **H** Results from the Western blot analysis of ets1, PFN2, CD31, vimentin, αSMA and S100A4 in the HUVECs with the corresponding treatment. **I** The effects of ets1 overexpression were confirmed by qPCR. **J** The effects of si-PFN2 were confirmed by qPCR. **K** ets1 overexpression inhibited CD31 mRNA expression, which was reversed by si-PFN2 treatment. **L** ets1 overexpression increased vimentin mRNA expression, which was reversed by si-PFN2 treatment. **M** ets1 overexpression increased αSMA mRNA expression, which was reversed by si-PFN2 treatment. **N** ets1 overexpression increased S100A4 mRNA expression, which was reversed by si-PFN2 treatment. (* p < 0.05, ** p < 0.01, *** p < 0.001, **** p < 0.0001, n = 5/group)

Studies have indicated that ets1 plays a crucial role in the progression of DN (Geng et al. 2019; Liu et al. 2011). Accordingly, high glucose upregulated ets1 protein and mRNA levels in HUVECs (Additional file 4: Figure S3A, B). To further confirm the effect of ets1 on high glucose-mediated PFN2 expression and EndMT, both loss-of-function and gain-of-function approaches were used in the present study. The effect of si-ets1 was confirmed in this study (Fig. 3A, B). si-ets1 reversed high glucose-induced PFN2 expression (Fig. 3A, C). Moreover, si-ets1 counteracted the high glucose-mediated reduction in CD31 (Fig. 3A, D) and augmented vimentin, αSMA and S100A4 (Fig. 3A, E–G) levels in HUVECs. Moreover, the effect of ets1 overexpression was similar to the effect of high-glucose treatment (Fig. 3H-N). To determine whether the effects of ets1 overexpression were acquired by augmenting PFN2 levels, we silenced PFN2 in ets1-upregulated HUVECs. Our data found that PFN2 silencing counteracted ets1 overexpression-mediated reduction in CD31 (Fig. 3H, K) and augmented vimentin, αSMA and S100A4 (Fig. 3H, L-N) expression in HUVECs. These data demonstrated that upregulated ets1 augmented PFN2 levels in hyperglycemic HUVECs, thus participating in high glucose-mediated EndMT in HUVECs.

### ets1 associated with KMT5A

**Fig. 4:**
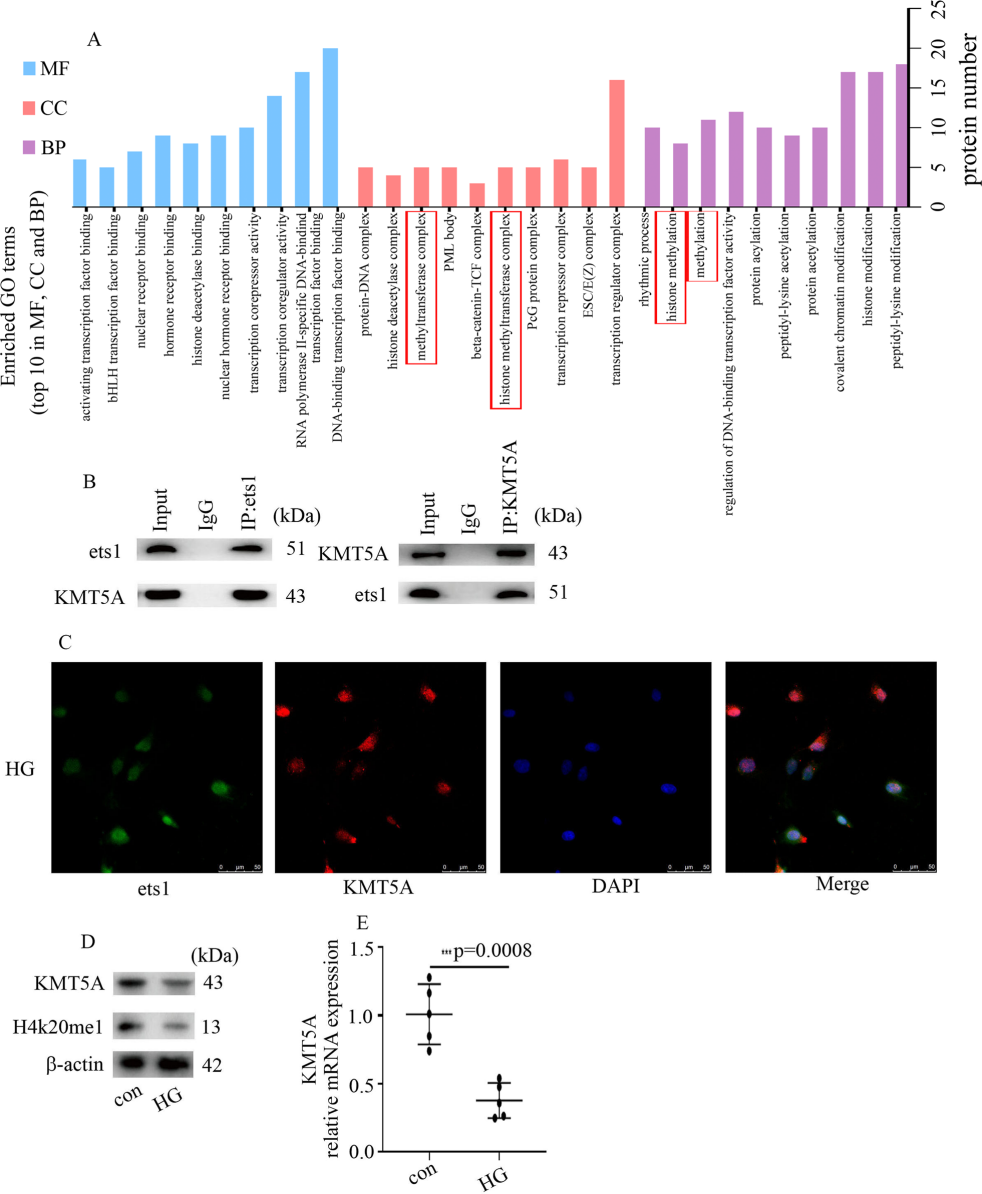
ets1 associated with KMT5A. **A** The enriched gene ontology (GO) terms. The vertical axis in the graph represents the number of significant proteins. The horizontal axes represent the enriched GO terms. (BP: biological processes; MF: molecular functions; CC: cellular components). **B** The association between ets1 and KMT5A in HUVECs was confirmed by co-IP. **C** Colocalization of ets1 and KMT5A in HUVECs was tested by confocal microscopy. **D** Results from the Western blot analysis of KMT5A and H4K20me1 in the HUVECs with the corresponding treatment. **E** Compared with the control group, the mRNA expression of KMT5A was decreased in hyperglycemic HUVECs. (* p < 0.05, ** p < 0.01, *** p < 0.001, **** p < 0.0001, n = 5/group)

To uncover the potential regulatory mechanism by which ets1 regulates PFN2 levels as well as EndMT in hyperglycemic HUVECs, bioinformatics was employed to predict the proteins that associate with ets1 in the present study. Many proteins that associated with ets1 are shown in Table 2, including KMT5A (https://inbio-discover.intomics.com/map.html#search). The results of GO pathway enrichment analysis showed that the top ten enriched GO terms in biological processes (BP), molecular functions (MF) and cellular components (CC) were arranged in Fig. 4A, including histone methylation which was highlighted by the red boxes. Our previous studies demonstrated that KMT5A downregulation participates in high glucose-mediated cell injury in HUVECs (Chen et al. 2018, 2020a, b; Qi et al. 2020; Wang et al. 2020; Shen et al. 2020). Moreover, KMT5A also mediates the occurrence of epithelial-to-mesenchymal transition (Hou et al. 2016). The association between ets1 and KMT5A in HUVECs was confirmed by CoIP experiments (Fig. 4B). IF experiments revealed that ets1 and KMT5A were co-positioned in the nucleus of hyperglycemic HUVECs (Fig. 4C). Furthermore, our data indicated that high-glucose treatment inhibited KMT5A levels (Fig. 4D, E) in HUVECs. Accordingly, the expression of H4K20me1, a downstream target of KMT5A, was inhibited in the HG group (Fig. 4D).

**Table 2:** Proteins which associate with ets1

Receptor	SRA1
Transcription factor	AEBP2 LEF1 NFKB2 ZBTB7A NCOR1 TWIST1 SMAD3 RUNX2 SP1 ATF2 SP100 PIAS4 PRDM1 MAF GFI1 TCF7L2 POU1F1 TCF4 SPI1 ETS2 JUN PAX5 ARID4B PARP1 RUNX1T1 TAL1 FOXP3 STAT6 FOXO1;
Kinase	CSNK1A1
Enzyme	***KMT5A*** EZH2 CREBBP COP1 NCOA3 NCOA1 TDP2 RBBP6 USP17L2 HDAC2
Other	DTL L3MBTL1 EED STRA8 SUZ12 DAXX TLX3 ZMIZ1 TLX1 MEIS1 CYBC1

SRAI (Steroid Receptor RNA Activator 1), AEBP2 (AE Binding Protein 2), LEF1 (Lymphoid Enhancer Binding Factor 1), NFKB2 (Nuclear Factor Kappa B Subunit 2), ZBTB7A (Zinc Finger And BTB Domain Containing 7A), NCOR1 (Nuclear Receptor Corepressor 1), TWIST1 (Twist Family BHLH Transcription Factor 1), SMAD3 (SMAD Family Member 3), RUNX2 (RUNX Family Transcription Factor 2), SP1 (Sp1 Transcription Factor), ATF2 (Activating Transcription Factor 2), SP100 (SP100 Nuclear Antigen), PIAS4 (Protein Inhibitor Of Activated STAT 4), PRDM1 (PR/SET Domain 1), MAF (MAF BZIP Transcription Factor), GFI1 (Growth Factor Independent 1 Transcriptional Repressor), TCF7L2 (Transcription Factor 7 Like 2), POU1F1 (POU Class 1 Homeobox 1), TCF4 (Transcription Factor 4), SPI1 (Spi-1 Proto-Oncogene), ETS2 (ETS Proto-Oncogene 2, Transcription Factor), JUN (Jun Proto-Oncogene, AP-1 Transcription Factor Subunit), PAX5 (Paired Box 5), ARID4B (AT-Rich Interaction Domain 4B), PARP1 (Poly(ADP-Ribose) Polymerase 1), RUNX1T1 (RUNX1 Partner Transcriptional Co-Repressor 1), TAL1 (TAL BHLH Transcription Factor 1, Erythroid Differentiation Factor), FOXP3 (Forkhead Box P3), STAT6 (Signal Transducer And Activator Of Transcription 6), FOXO1 (Forkhead Box O1), CSNK1A1 (Casein Kinase 1 Alpha 1), *KMT5A (Lysine Methyltransferase 5A)*, EZH2 (Enhancer Of Zeste 2 Polycomb Repressive Complex 2 Subunit), CREBBP (CREB Binding Protein), COP1 (COP1 E3 Ubiquitin Ligase), NCOA3 (Nuclear Receptor Coactivator 3), TDP2 (Tyrosyl-DNA Phosphodiesterase 2), RBBP6 (RB Binding Protein 6, Ubiquitin Ligase), USP17L2 (Ubiquitin Specific Peptidase 17 Like Family Member 2), HDAC2 (Histone Deacetylase 2), DTL (Denticleless E3 Ubiquitin Protein Ligase Homolog), L3MBTL1 (L3MBTL Histone Methyl-Lysine Binding Protein 1), EED (Embryonic Ectoderm Development), STRA8 (Stimulated By Retinoic Acid 8), SUZ12 (SUZ12 Polycomb Repressive Complex 2 Subunit), DAXX (Death Domain Associated Protein), TLX3 (T Cell Leukemia Homeobox 3), ZMIZ1 (Zinc Finger MIZ-Type Containing 1), TLX1 (T Cell Leukemia Homeobox 1), MEIS1 (Meis Homeobox 1), CYBC1 (Cytochrome B-245 Chaperone 1)

### KMT5A suppression participated in high glucose-mediated EndMT by augmenting PFN2 levels in HUVECs

**Fig. 5:**
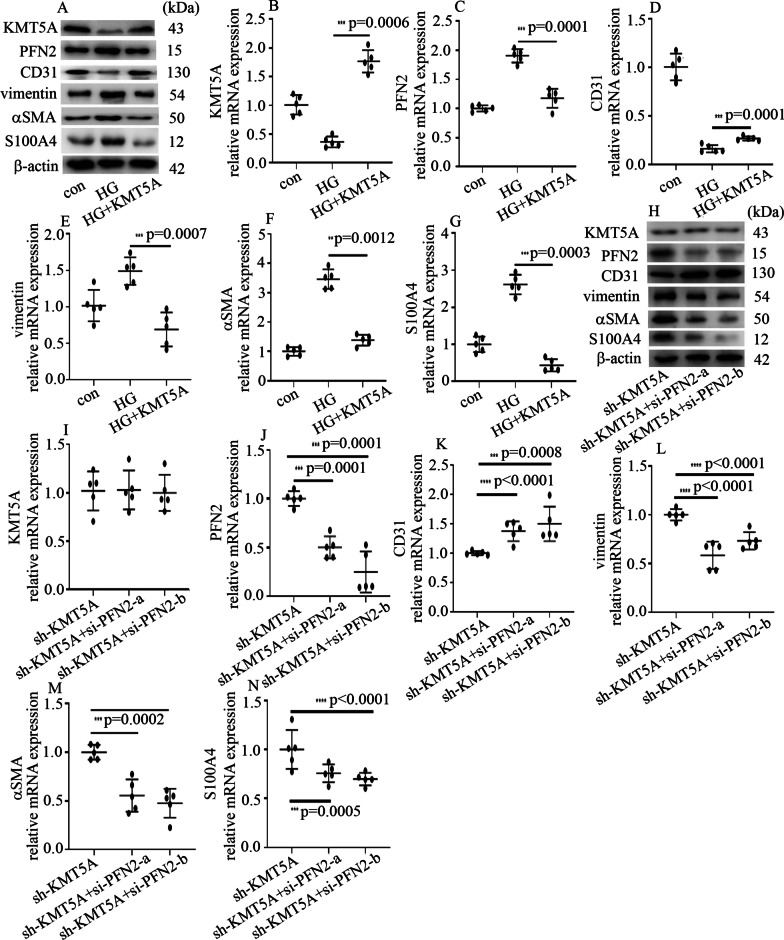
KMT5A suppression participated in high glucose-mediated EndMT by augmenting PFN2 expression in HUVECs. **A** Results from the Western blot analysis of KMT5A, PFN2, CD31, vimentin, αSMA and S100A4 in the HUVECs with the corresponding treatment. **B** The effects of KMT5A overexpression were verified by qPCR. **C** The high glucose-mediated increase in PFN2 mRNA expression was inhibited by KMT5A overexpression. **D** The high glucose-mediated decrease in CD31 mRNA expression was counteracted by KMT5A overexpression. **E** The high glucose-mediated increase in vimentin mRNA expression was inhibited by KMT5A overexpression. **F** The high glucose-mediated increase in αSMA mRNA expression was inhibited by KMT5A overexpression. **G** The high glucose-mediated increase in S100A4 mRNA expression was inhibited by KMT5A overexpression. **H** Results from the Western blot analysis of KMT5A, PFN2, CD31, vimentin, αSMA and S100A4 in the HUVECs with the corresponding treatment. **I** si-PFN2 did not affect KMT5A mRNA expression. **J** The effect of si-PFN2 was verified by qPCR. **K** si-PFN2 upregulated CD31 mRNA expression in sh-KMT5A-treated HUVECs. **L** si-PFN2 decreased vimentin mRNA expression in sh-KMT5A-treated HUVECs. **M** si-PFN2 decreased αSMA mRNA expression in sh-KMT5A-treated HUVECs. **N** si-PFN2 decreased S100A4 mRNA expression in sh-KMT5A-treated HUVECs. (* p < 0.05, ** p < 0.01, *** p < 0.001, **** p < 0.0001, n = 5/group)

To determine whether KMT5A modulates PFN2 levels and EndMT in hyperglycemic HUVECs, both loss-of-function and gain-of-function approaches were employed in the present study. The effect of KMT5A overexpression was verified in the present study (Fig. 5A, B). The results demonstrated that KMT5A overexpression reversed high glucose-induced PFN2 (Fig. 5A, C). Moreover, KMT5A overexpression counteracted the high glucose-mediated reduction in CD31 (Fig. 5A, D) and augmented vimentin, αSMA and S100A4 (Fig. 3A, E–G) levels in HUVECs.

The effect of sh-KMT5A was verified in the present study (Additional file 5: Figure S4A, B). The effect of sh-KMT5A was similar to the effect of high-glucose treatment (Additional file 5: Figure S4A, C-G). To determine whether the effects of sh-KMT5A were acquired via the augmentation of PFN2 expression, PFN2 was silenced in HUVECs in which KMT5A was downregulated. Our data indicated that PFN2 silencing counteracted the KMT5A downregulation-mediated EndMT in HUVECs (Fig. 5H-N). These data indicated that KMT5A downregulation augmented PFN2 levels, thus participating in high glucose-mediated EndMT in HUVECs.

### ets1 cooperated with KMT5A to regulate PFN2 transcriptional activity in HUVECs

**Fig. 6:**
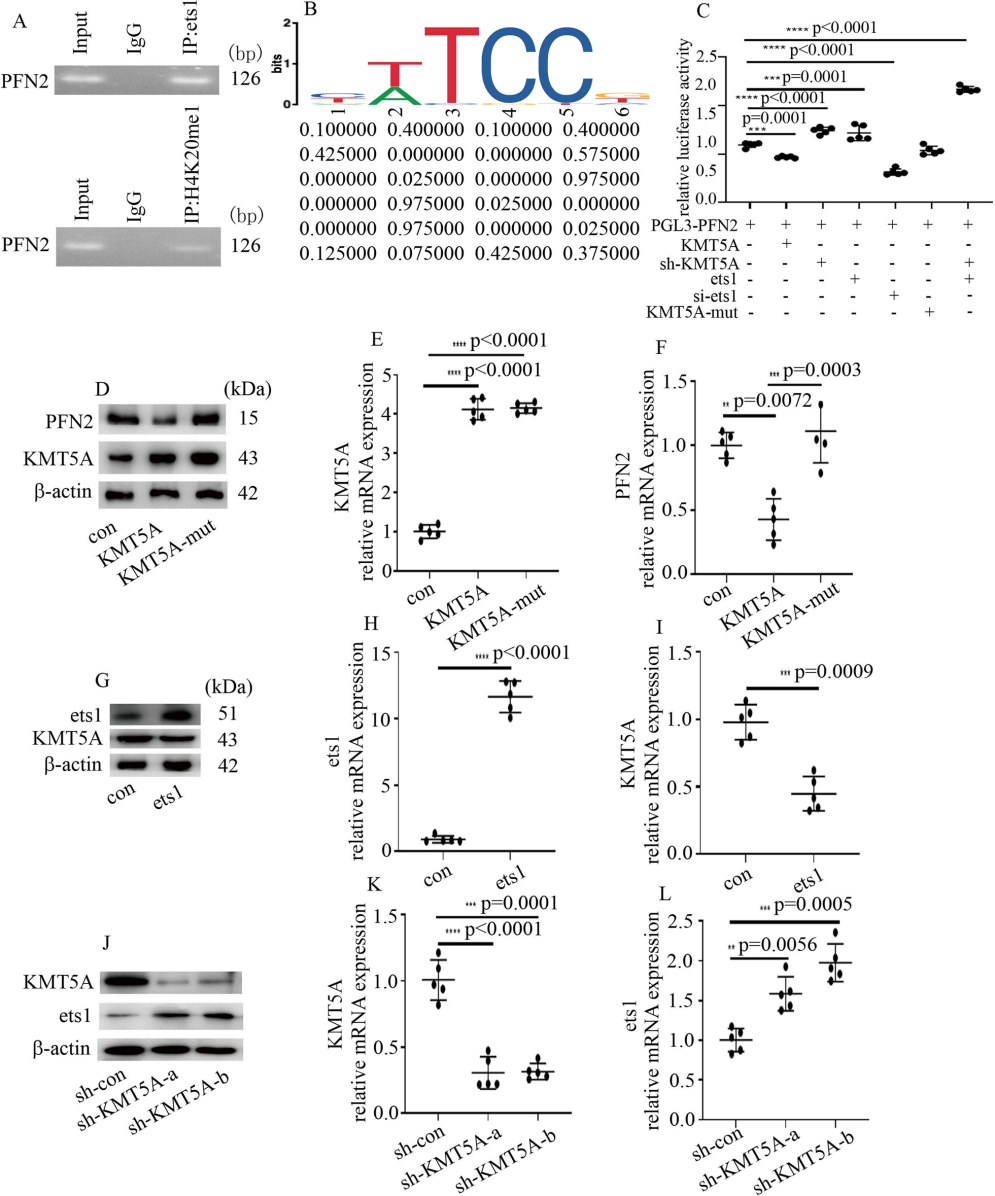
ets1 cooperated with KMT5A to regulate PFN2 transcriptional activity in HUVECs. **A** ets1 and H4K20me1 were enriched at the PFN2 promoter region. **B** The putative ets1 binding site of PFN2. The motif logo and position weight matrix are shown in the upper and lower panels, respectively. **C** PFN2 promoter activity was determined by luciferase reporter assays with the corresponding treatment. **D** Results from the Western blot analysis of KMT5A and PFN2 in the HUVECs with the corresponding treatment. **E** Compared with the control group, the KMT5A overexpression and mutant KMT5A^R259G^ plasmids both increased KMT5A mRNA expression in HUVECs. **F** Compared with the control group, KMT5A overexpression decreased PFN2 mRNA expression, while mutant KMT5A^R259G^ did not affect PFN2 mRNA expression. **G** Results from the Western blot analysis of KMT5A and ets1 in HUVECs with the corresponding treatment. **H** ets1 overexpression was confirmed by qPCR. **I** Compared with the control group, ets1 overexpression decreased KMT5A mRNA expression in HUVECs. **J** Results from the Western blot analysis of KMT5A and ets1 in the HUVECs with the corresponding treatment. **K** The effects of sh-KMT5A were confirmed by qPCR. **L** sh-KMT5A increased ets1 mRNA expression in HUVECs. (* p < 0.05, ** p < 0.01, *** p < 0.001, **** p < 0.0001, n = 5/group)

To explore whether PFN2 is directly transcribed by ets1 and KMT5A, the genome-wide distribution of ets1 and H4K20me1 was detected in the present study. ChIP assays demonstrated that ets1 and H4K20me1 both occupied the PFN2 promoter region (Fig. 6A). The potential ets1 binding site is exhibited in Fig. 6B. Moreover, KMT5A overexpression and si-ets1 both decreased PFN2 promoter activity (Fig. 6C). sh-KMT5A and ets1 overexpression both augmented PFN2 promoter activity (Fig. 6C). Moreover, sh-KMT5A acted in concert with ets1 overexpression to augment PFN2 promoter activity in HUVECs (Fig. 6C). However, mutant KMT5A^R259G^ had no effect on PFN2 promoter activity (Fig. 6C). These data indicated that ets1 cooperated with KMT5A to modulate PFN2 promoter activity in hyperglycemic HUVECs. Furthermore, KMT5A overexpression inhibited PFN2 protein and mRNA levels, while mutant KMT5A^R259G^ did not affect PFN2 levels (Fig. 6D-F). Our data indicated that KMT5A-mediated H4K20me1 was necessary to modulate the PFN2 transcript in HUVECs. Furthermore, ets1 overexpression decreased KMT5A expression (Fig. 6G-I). Accordingly, sh-KMT5A augmented ets1 expression in HUVECs (Fig. 6J-L). Our study showed that ets1 and KMT5A inhibited each other in HUVECs.

### High glucose-mediated increases in ets1 and decreases in KMT5A were verified in DN participants and rats

**Fig. 7:**
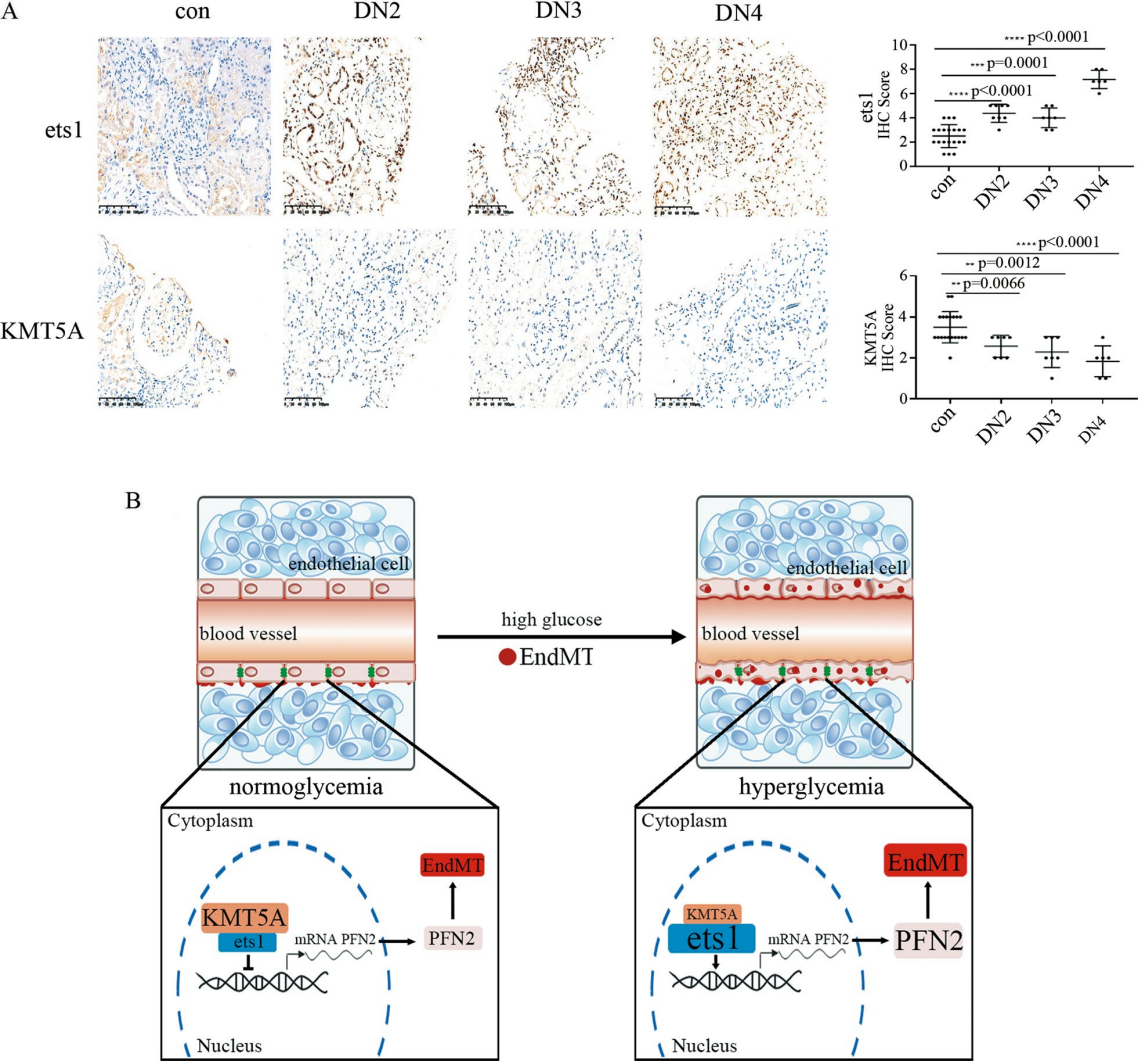
High glucose-mediated increases in ets1 and decreases in KMT5A were verified in DN patients and rats. **A** Immunostaining of ets1 and KMT5A in renal biopsy specimens of DN patients and control participants. Magnification: ×20. Scale bar: 20 μM. (* p < 0.05, ** p < 0.01, *** p < 0.001, **** p < 0.0001, n = 20 for the control group, n = 7 for the DN2 group, n = 7 for the DN3 group, n = 6 for the DN4 group). **B** Schematic representation of the working model

To explore whether the protein and/or mRNA expression of ets1 and KMT5A in DN participants and rats was consistent with the protein and/or mRNA expression of ets1 and KMT5A of the in vitro study, ets1 and KMT5A levels were assessed in kidney tissues and/or aortic tissues of DN patients and rats. The results indicated that ets1 increased and KMT5A decreased in glomerular endothelial cells of DN patients (Fig. 7A) and rats (Additional file 6: Figure S5A). More importantly, our data indicated that ets1 levels gradually increased, while KMT5A gradually decreased in glomeruli of DN patients when the DN stage progressed (Fig. 7A). Accordingly, the expression of ets1 increased, while the expression of KMT5A decreased in aortic tissues of DN rats (Additional file 6: Figure S5B-D). In conclusion, our study showed that ets1 and KMT5A cooperated to regulate PFN2 transcription, thus mediating EndMT in DN patients and rats (Fig. 7B).

## Discussion

The main finding of this study was that high glucose participated in EndMT via the augmentation of PFN2 transcription, thus mediating endothelial cell injury and the occurrence of DN. Moreover, high-glucose treatment decreased KMT5A levels and augmented ets1 levels. Furthermore, ets1 and H4K20me1 both occupied the PFN2 promoter region. Mechanistic studies indicated that KMT5A associates with ets1 to regulate PFN2 transcriptional activity, thus mediating EndMT in hyperglycemic HUVECs.

Epithelial-to-mesenchymal transition is a complicated form of cell phenotype reprogramming and plays a crucial role in organ injury (Duan et al. 2012). The epithelial-to-mesenchymal transition of renal tubular epithelial cells plays an important role in renal interstitial myofibroblasts, thus participating in the progression of renal fibrosis (Stasi et al. 2017). Recently, EndMT of glomerular endothelial cells was proven to participate in the occurrence and progression of DN (Li et al. 2010; Kanasaki et al. 2014). In the present study, the results indicated that CD31 was reduced, while vimentin, αSMA and S100A4 were augmented in the glomerular endothelial cells of DN participants and rats (Fig. 1, Additional file 2: Figure S1). Moreover, HE staining and Masson trichrome staining indicated renal injury and interstitial fibrosis in the glomeruli of DN participants and rats (Fig. 1, Additional file 2: Figure S1). These data were in agreement with a recent study that indicated EndMT in glomeruli aggravates DN (Peng et al. 2016). To determine whether EndMT in DN patients and rats was caused by hyperglycemia, we used high glucose-treated HUVECs as an in vitro model. The protein and mRNA levels of CD31, vimentin, αSMA and S100A4 confirmed that high glucose participated in the occurrence of EndMT, which was similar to one previous study (Yu et al. 2017). We deduced that epithelial-to-mesenchymal transition and EndMT may have shared modulators (Saito 2013). PFN2 has been reported to participate in epithelial-to-mesenchymal transition (Tang et al. 2015; Zhang et al. 2018). In the present study, PFN2 levels were found to be increased in DN patients and rats (Fig. 1). Moreover, high-glucose treatment induced PFN2 expression (Fig. 2A, F) and EndMT (Fig. 2A-E) in HUVECs. The inhibition of PFN2 expression reversed high glucose-mediated EndMT (Fig. 2G-L). Our data indicated that PFN2 was involved in high glucose-mediated EndMT in HUVECs. In diabetic nephropathy or high-glucose conditions, several other types of cells undergo damage, including podocytes and tubular epithelial cells. Previous studies have demonstrated that crosstalk between tubular epithelial cells and glomerular endothelial cells plays an important role in diabetic nephropathy (Chen et al. 2020). Exosomes from high glucose-treated glomerular endothelial cells have been reported to trigger epithelial-mesenchymal transition and dysfunction in podocytes (Wu et al. 2017). These studies indicated that endothelial cells, podocytes, and tubular epithelial cells interact with each other, and all participate in the progression of diabetic nephropathy.

ets1, a member of the ETS family of transcription factors, plays a crucial role in kidney development and glomerular integrity (Razzaque et al. 2001). The expression of ets1 has been reported to increase in both glomeruli and interstitium during the progression of rat crescentic glomerulonephritis (Naito et al. 2000). Moreover, ets1 serves as a vital transcription factor in the progression of DN (Geng et al. 2019). ets1 upregulation was reported to participate in glomerulosclerosis and renal interstitial fibrosis in experimental DN (Liu et al. 2011). Similarly, ets1 suppression protects against angiotensin II-mediated cardiac fibrosis via inhibition of EndMT (Xu et al. 2019). The mechanism by which ets1 modulates EndMT involves the transactivation of Twist1 promoter activity (Millien et al. 2018) and the activation of the ZEB1 promoter (Sinh et al. 2017; Dave et al. 2011). The present study showed that high glucose augmented ets1 expression in HUVECs (Additional file 4: Figure S3A, B). Moreover, si-ets1 reversed high glucose-mediated PFN2 upregulation and EndMT (Fig. 3A-G). Furthermore, ets1 overexpression increased PFN2 expression and mediated EndMT, which was counteracted by si-PFN2 (Fig. 3H-N). These data indicated that ets1 was involved in high glucose-mediated EndMT in hyperglycemic HUVECs via the augmentation of PFN2 expression.

Our previous studies showed that KMT5A participates in high glucose-mediated overexpression of endothelial adhesion molecule expression (Chen et al. 2018; Shen et al. 2020), proinflammatory enzyme and proinflammatory cytokine production (Qi et al. 2020), antioxidant imbalance (Chen et al. 2020) and NOD-like receptor pyrin domain 3 inflammasome activation (Wang et al. 2020) in endothelial cells, thus mediating vascular endothelial injury (Chen et al. 2018, 2020a, b; Qi et al. 2020; Wang et al. 2020; Shen et al. 2020). However, the regulation of EndMT by KMT5A has not been reported. Indeed, KMT5A has been reported to be involved in epithelial-to-mesenchymal transition via the modulation of TWIST (Yang et al. 2012) and cooperation with zinc finger E-box-binding homeobox 1 (Hou et al. 2016). As a specific form of epithelial-to-mesenchymal transition, it is possible that EndMT and epithelial-to-mesenchymal transition share mutual modulators (Saito 2013). In the present study, KMT5A overexpression counteracted high glucose-mediated PFN2 upregulation (Fig. 5A, C) and EndMT (Fig. 3A, D-G) in HUVECs. Moreover, H4K20me1, a direct target of KMT5A, occupied the PFN2 promoter region (Fig. 6A). Furthermore, si-PFN2 counteracted sh-KMT5A-mediated EndMT (Fig. 5H-N). These data indicated that high glucose-mediated KMT5A downregulation participated in EndMT in hyperglycemic HUVECs by augmenting PFN2 expression. KMT5A is the only known H4K20me1 regulator, but H4K20me1 is not the only KMT5A target (Beck et al. 2012). In our further research, one way to assess the EndMT-specific effects of KMT5A downregulation in hyperglycemic HUVECs would be to employ gene expression microarrays and explore the expression status of EndMT-specific genes.

The transcriptional activity of ets1 has been reported to be regulated by epigenetic modifications (Liu et al. 2020; Sobral et al. 2020). The present study represented an association between KMT5A and ets1 (Fig. 4B, C). Moreover, ets1 and H4K20me1 both occupied the PFN2 promoter region (Fig. 6A). Furthermore, sh-KMT5A augmented the promoting action of ets1 on PFN2 promoter activity (Fig. 6C). The results showed that KMT5A cooperated with ets1 to adjust PFN2 transcription, thus participating in EndMT in high glucose-treated HUVECs (Fig. 7C). Furthermore, KMT5A upregulation reduced PFN2 levels (Fig. 6D, F). However, KMT5A^R259G^ (one KMT5A mutant) had no effect on PFN2 transcription (Fig. 6D, F). Our study showed that KMT5A-induced H4K20me1 participated in the regulation of PFN2 transcription.

This study has some limitations. First, whether KMT5A associates with ets1 in a direct or indirect manner is worthy of further study. Second, HUVECs were used to establish an in vitro model, and other primary endothelial cells should be used to verify our results in the present study. Third, the mechanism of mutual suppression between KMT5A and ets1 deserves further research. Fourth, the potential mechanism by which PFN2 enhances EndMT in hyperglycemic HUVECs was not well investigated in this study. Previous studies have indicated that vasodilator-stimulated phosphoprotein (VASP) (Reinhard et al. 1995) serves as a partner of PFN2, and VASP has been reported to participate in epithelial-mesenchymal transition (Zhang et al. 2020). Whether PFN2 participates in EndMT via association with VASP or other partners deserves further research. Fifth, the number of cases in each DN group is limited, so the present study needs more cases to be further confirmed.

## Conclusions

Our data indicated that KMT5A levels were reduced, ets1 and PFN2 levels were upregulated, and EndMT was induced in DN patients and rats. The present study also demonstrated that high glucose induced EndMT by augmenting PFN2 levels in hyperglycemic HUVECs. Moreover, high glucose levels suppressed KMT5A levels and increased ets1 levels in vascular endothelial cells. Furthermore, KMT5A cooperated with ets1 to modulate PFN2 levels, thus participating in high glucose-mediated EndMT in hyperglycemic HUVECs.

## Supplementary Information

**Additional file 1: Table S1.** Primers used for real-time qPCR analysis.

**Additional file 2: Figure S1.** Occurrence of EndMT and increase in PFN2expression in the glomeruli of DN rats. (A) HE staining and Masson stainingof renal biopsy specimens from DN rats and control rats. Magnification:40 × . Scale bar: 10 μM. (B) Immunostaining of CD31, vimentin, αSMA,S100A4 and PFN2 in renal biopsy specimens of DN rats and control rats.Magnification: 40 × . Scale bar: 10 μM. (* p < 0.05, ** p < 0.01, *** p < 0.001,**** p < 0.0001, n = 10/group).

**Additional file 3: Figure S2.** Occurrence of EndMT and increase in PFN2expression in aortic tissues of DN rats. (A) Immunostaining of CD31,vimentin, αSMA, S100A4 and PFN2 in aortic tissues of DN rats and controlrats. (B) Compared with the control group, the mRNA expression of CD31was decreased in aortic tissues of DN rats. (C) Compared with the controlgroup, the mRNA expression of vimentin was increased in aortic tissuesof DN rats. (D) Compared with the control group, the mRNA expressionof αSMA was increased in aortic tissues of DN rats. (E) Compared with thecontrol group, the mRNA expression of S100A4 was increased in aortictissues of DN rats. (F) Compared with the control group, the mRNA expressionof PFN2 was increased in aortic tissues of DN rats. (G) Blood glucoseevolution in the rats started after the induction of diabetes at 6 weeks.Magnification: 20 × . Scale bar: 20 μM. (* p < 0.05, ** p < 0.01, *** p < 0.001,**** p < 0.0001, n = 10/group).

**Additional file 4: Figure S3.** High glucose upregulated ets1 expressionin the HUVECs. (A) Results from the Western blot analysis of ets1 in theHUVECs with the corresponding treatment. (B) Compared with the controlgroup, the mRNA expression of ets1 was increased in hyperglycemicHUVECs. (* p < 0.05, ** p < 0.01, *** p < 0.001, **** p < 0.0001, n = 5/group).

**Additional file 5: Figure S4.** sh-KMT5A induced EndMT and PFN2 expression inHUVECs. (A) Results from the Western blot analysis of KMT5A, PFN2, CD31,vimentin, αSMA, and S100A4 in the HUVECs with the corresponding treatment.(B) The effects of sh-KMT5A were confirmed by qPCR. (C) Comparedwith the control group, sh-KMT5A increased PFN2 mRNA expression inHUVECs. (D) Compared with the control group, sh-KMT5A decreasedCD31 mRNA expression in HUVECs. (E) Compared with the control group,sh-KMT5A increased vimentin mRNA expression in HUVECs. (F) Comparedwith the control group, sh-KMT5A increased αSMA mRNA expressionin HUVECs. (G) Compared with the control group, sh-KMT5A increasedS100A4 mRNA expression in HUVECs. (* p < 0.05, ** p < 0.01, *** p < 0.001,**** p < 0.0001, n = 5/group).

**Additional file 6: Figure S5.** The expression of ets1 and KMT5A in DN ratsand control rats. (A) Immunostaining of ets1 and KMT5A in renal biopsyspecimens of DN rats and control rats. (Magnification: 40 × . Scale bar:10 μM) (B) Immunostaining of ets1 and KMT5A in aortic tissues of DN ratsand control rats. (Magnification: 20 × . Scale bar: 20 μM) (C) Comparedwith the control group, the mRNA expression of ets1 was increased inaortic tissues of DN rats. (D) Compared with the control group, the mRNAexpression of KMT5A was decreased in aortic tissues of DN rats. (* p < 0.05,** p < 0.01, *** p < 0.001, **** p < 0.0001, n = 10/group).

## Data Availability

The datasets used and/or analysed during the current study are available from the corresponding author on reasonable request.

## Abbreviations

DN: Diabetic nephropathy
EndMT: Endothelial-to-mesenchymal transition
KMT5A: Lysine Methyltransferase 5A
HUVECs: Human umbilical vein endothelial cells
H4K20me1: Histone H4 lysine20 methylation
ets1: ETS proto-oncogene 1
PFN2: Profilin2

## References

[CR1] Alves TP, Lewis J (2010). Racial differences in chronic kidney disease (CKD) and end-stage renal disease (ESRD) in the United States: a social and economic dilemma. Clin Nephrol.

[CR2] Beck DB, Oda H, Shen SS, Reinberg D (2012). PR-Set7 and H4K20me1: at the crossroads of genome integrity, cell cycle, chromosome condensation, and transcription. Genes Dev.

[CR3] Boer IH, Rue TC, Hall YN, Heagerty PJ, Weiss NS, Himmelfarb J (2011). Temporal trends in the prevalence of diabetic kidney disease in the United States. JAMA.

[CR4] Chen X, Wu Q, Jiang H, Wang J, Zhao Y, Xu Y (2018). SET8 is involved in the regulation of hyperglycemic memory in human umbilical endothelial cells. Acta Biochim Biophys Sin.

[CR5] Chen X, Qi J, Wu Q, Jiang H, Wang J, Chen W (2020). High glucose inhibits vascular endothelial Keap1/Nrf2/ARE signal pathway via downregulation of monomethyltransferase SET8 expression. Acta Biochim Biophys Sin.

[CR6] Chen SJ, Lv LL, Liu BC, Tang RN (2020). Crosstalk between tubular epithelial cells and glomerular endothelial cells in diabetic kidney disease. Cell Prolif..

[CR7] Dave N, Guaita-Esteruelas S, Gutarra S, Frias À, Beltran M, Peiró S (2011). Functional cooperation between Snail1 and twist in the regulation of ZEB1 expression during epithelial to mesenchymal transition. J Biol Chem.

[CR8] Ding Z, Bae YH, Roy P (2012). Molecular insights on context-specific role of profilin-1 in cell migration. Cell Adh Migr.

[CR9] Duan SB, Liu GL, Wang YH, Zhang JJ (2012). Epithelial-to-mesenchymal transdifferentiation of renal tubular epithelial cell mediated by oxidative stress and intervention effect of probucol in diabetic nephropathy rats. Ren Fail.

[CR10] Geng XD, Wang WW, Feng Z, Liu R, Cheng XL, Shen WJ (2019). Identification of key genes and pathways in diabetic nephropathy by bioinformatics analysis. J Diabetes Investig.

[CR11] Hou L, Li Q, Yu Y, Li M, Zhang D (2016). SET8 induces epithelial-mesenchymal transition and enhances prostate cancer cell metastasis by cooperating with ZEB1. Mol Med Rep.

[CR12] Kanasaki K, Shi S, Kanasaki M, He J, Nagai T, Nakamura Y (2014). Linagliptin-mediated DPP-4 inhibition ameliorates kidney fibrosis in streptozotocin-induced diabetic mice by inhibiting endothelial-to-mesenchymal transition in a therapeutic regimen. Diabetes.

[CR13] Li J, Qu X, Yao J, Caruana G, Ricardo SD, Yamamoto Y (2010). Blockade of endothelialmesenchymal transition by a Smad3 inhibitor delays the early development of streptozotocin-induced diabetic nephropathy. Diabetes.

[CR14] Liang X, Duan N, Wang Y, Shu S, Xiang X, Guo T (2016). Advanced oxidation protein products induce endothelial-to-mesenchymal transition in human renal glomerular endothelial cells through induction of endoplasmic reticulum stress. J Diabetes Compl.

[CR15] Liu D, Liu X, Su Y, Zhang X (2011). Renal expression of protooncogene Ets-1 on matrix remodeling in experimental diabetic nephropathy. Acta Histochem.

[CR16] Liu J, Li D, Zhang X, Li Y, Ou J (2020). Histone Demethylase KDM3A Promotes Cervical Cancer Malignancy Through the ETS1/KIF14/Hedgehog Axis. Onco Targets Ther.

[CR17] Millien G, Cao Y, O'Hara CJ, Tagne JB, Hinds A, Williams MC (2018). ETS1 regulates Twist1 transcription in a Kras (G12D) /Lkb1(-/-) metastatic lung tumor model of non-small cell lung cancer. Clin Exp Metastasis.

[CR18] Naito T, Razzaque MS, Nazneen A, Liu D, Nihei H, Koji T (2000). Renal expression of the Ets-1 proto-oncogene during progression of rat crescentic glomerulonephritis. J Am Soc Nephrol.

[CR19] Ng KP, Jain P, Gill PS, Heer G, Townend JN, Freemantle N (2016). Results and lessons from the spironolactone to prevent cardiovascular events in early stage chronic kidney disease (STOP-CKD) randomized controlled trial. BMJ Open..

[CR20] Packham DK, Alves TP, Dwyer JP, Atkins R, Zeeuw D, Cooper M (2012). Relative incidence of ESRD versus cardiovascular mortality in proteinuric type 2 diabetes and nephropathy: results from the DIAMETRIC (Diabetes Mellitus Treatment for Renal Insufficiency Consortium) database. Am J Kidney Dis.

[CR21] Peng H, Li Y, Wang C, Zhang J, Chen Y, Chen W (2016). ROCK1 induces endothelial-to-mesenchymal transition in glomeruli to aggravate albuminuria in diabetic nephropathy. Sci Rep.

[CR22] Qi J, Wu Q, Cheng Q, Chen X, Zhu M, Miao C (2020). High glucose induces endothelial COX2 and iNOS expression via inhibition of monomethyltransferase SETD8 expression. J Diabetes Res.

[CR23] Razzaque MS, Naito T, Taguchi T (2001). Proto-oncogene ets-1 and the kidney. Nephron.

[CR24] Reinhard M, Giehl K, Abel K, Haffner C, Jarchau T, Hoppe V (1995). The proline-rich focal adhesion and microfilament protein VASP is a ligand for profilins. EMBO J.

[CR25] Saito A (2013). EMT and EndMT: regulated in similar ways. Comment J Biochem.

[CR26] Shen X, Chen X, Wang J, Liu J, Wang Z, Hua Q (2020). SET8 suppression mediates high glucose-induced vascular endothelial inflammation via the upregulation of PTEN. Exp Mol Med.

[CR27] Sinh ND, Endo K, Miyazawa K, Saitoh M (2017). Ets1 and ESE1 reciprocally regulate expression of ZEB1/ZEB2, dependent on ERK1/2 activity, in breast cancer cells. Cancer Sci.

[CR28] Sobral LM, Hicks HM, Parrish JK, McCann TS, Hsieh J, Goodspeed A (2020). KDM3A/Ets1 epigenetic axis contributes to PAX3/FOXO1-driven and independent disease-promoting gene expression in fusion-positive Rhabdomyosarcoma. Mol Oncol.

[CR29] Stasi A, Intini A, Divella C, Franzin R, Montemurno E, Grandaliano G (2017). Emerging role of Lipopolysaccharide binding protein in sepsis-induced acute kidney injury. Nephrol Dial Transplant.

[CR30] Tang YN, Ding WQ, Guo XJ, Yuan XW, Wang DM, Song JG (2015). Epigenetic regulation of Smad2 and Smad3 by profilin-2 promotes lung cancer growth and metastasis. Nat Commun.

[CR31] Tomino Y, Gohda T (2015). The prevalence and management of diabetic nephropathy in Asia. Kidney Dis.

[CR32] Wang J, Shen X, Liu J, Chen W, Wu F, Wu W (2020). High glucose mediates NLRP3 inflammasome activation via upregulation of ELF3 expression. Cell Death Dis.

[CR33] Wu X, Gao Y, Xu L, Dang W, Yan H, Zou D (2017). Exosomes from high glucose-treated glomerular endothelial cells trigger the epithelial-mesenchymal transition and dysfunction of podocytes. Sci Rep.

[CR34] Xu L, Fu M, Chen D, Han W, Ostrowski MC, Grossfeld P (2019). Endothelial-specific deletion of Ets-1 attenuates Angiotensin II-induced cardiac fibrosis via suppression of endothelial-to-mesenchymal transition. BMB Rep.

[CR35] Xue R, Gui D, Zheng L, Zhai R, Wang F, Wang N (2017). Mechanistic insight and management of diabetic nephropathy: recent progress and future perspective. J Diabetes Res.

[CR36] Yang F, Sun L, Li Q, Han X, Lei L, Zhang H (2012). SET8 promotes epithelial-mesenchymal transition and confers TWIST dual transcriptional activities. EMBO J.

[CR37] Yu CH, Gong M, Liu WJ, Cui NX, Wang Y (2017). High glucose induced endothelial to mesenchymal transition in human umbilical vein endothelial cell. Exp Mol Pathol..

[CR38] Zhang H, Yang W, Yan J, Zhou K, Wan B, Shi P (2018). Loss of profilin 2 contributes to enhanced epithelial-mesenchymal transition and metastasis of colorectal cancer. Int J Oncol.

[CR39] Zhang H, Cui X, Cao A, Li X, Li L (2020). ITGA3 interacts with VASP to regulate stemness and epithelial-mesenchymal transition of breast cancer cells. Gene..

