# Supplementary material for: ets1 associates with KMT5A to participate in high glucose-mediated EndMT via upregulation of PFN2 expression in diabetic nephropathy
Source: Mol Med. 2021 Jul 8;27:74. doi: 10.1186/s10020-021-00339-7 (PMC8266168; doi:10.1186/s10020-021-00339-7)
Supplement: Supplementary file 1 — Additional file 1: Table S1. Primers used for real-time qPCR analysis. [file 10020_2021_339_MOESM1_ESM.docx]

| species | RNA sequence |
| --- | --- |
| Human  β-actin  KMT5A  ets1  PFN2  vimentin  CD31  S100A4  αSMA  Rat  β-actin  KMT5A  ets1  PFN2  CD31  vimentin    S100A4  αSMA | F 5’- CGGCTACAGCTTCACCACCAC -3’  R 5’- GCCATCTCTTGCTCGAAGTCCAG -3’  F 5’- TCCAGCAATCCTCCTCCTTCCTC -3’  R 5’- CCAGCCTAAGCAACAGATCCAGA -3’  F 5’- TGGAGTCAACCCAGCCTATC -3’  R 5’- TCTGCAAGGTGTCTGTCTGC-3’  F 5’-GTAGGAAAAGACCGGGAAGG -3’  R 5’- GACACCTTCCTTTCCCATGA -3’  F 5’- TACACAATTGCCTCTCCCCC -3’  R 5’- ACTCCTGTCTGAGATTACCCT -3’  F 5’- ACAGGACCGCGTTTTATCCTT -3’  R 5’- CCTTCCCAGTTCTGGGTTCTT -3’  F 5’- CCACAAGTACTCGGGCAAAG -3’  R 5’- ATGCAGGACAGGAAGACACA -3’  F 5’-ACCCAGCACCATGAAGATCA-3’  R 5’- TTTGCGGTGGACAATGGAAG -3’  F 5’- CTTCCAGCCTTCCTTCCTGG -3’  R 5’- GAGCCACCAATCCACACAGA -3’  F 5’- GCAGGAAGAGAACTCCGTCG -3’  R 5’- AGAATCACATGACGGGGGTG -3’  F 5’- GAAATGATGTCCCAGGCACT -3’  R 5’- CTTTACCCAGGGCACACAGT-3’  F 5’- GAAAAGACCGGGAAGGTTTC -3’  R 5’-CAAGACTCTCCCAGCTCTGC -3’  F 5’- AGGCTGCCCTCAAACTCATC -3’  R 5’- CCCAACACGGATGCAAAAGG -3’  F 5’- GCCCCACCCTGAACCTAAAC-3’  R 5’- TGCCCTTCCCAACAATCACA-3’  F 5’- GGAGAAGGACAGACGAAGCT-3’  R 5’- TTCTTCCGGGGCTCCTTATC-3’  F 5’- CATCATGCGTCTGGACTTGG -3’  R 5’- CCAGGGAAGAAGAGGAAGCA-3’ |

Supplementary Table 1 Primers used for real-time qPCR analysis.
