# Supplementary figures and images for: ets1 associates with KMT5A to participate in high glucose-mediated EndMT via upregulation of PFN2 expression in diabetic nephropathy
Source: Mol Med. 2021 Jul 8;27:74. doi: 10.1186/s10020-021-00339-7 (PMC8266168; doi:10.1186/s10020-021-00339-7)

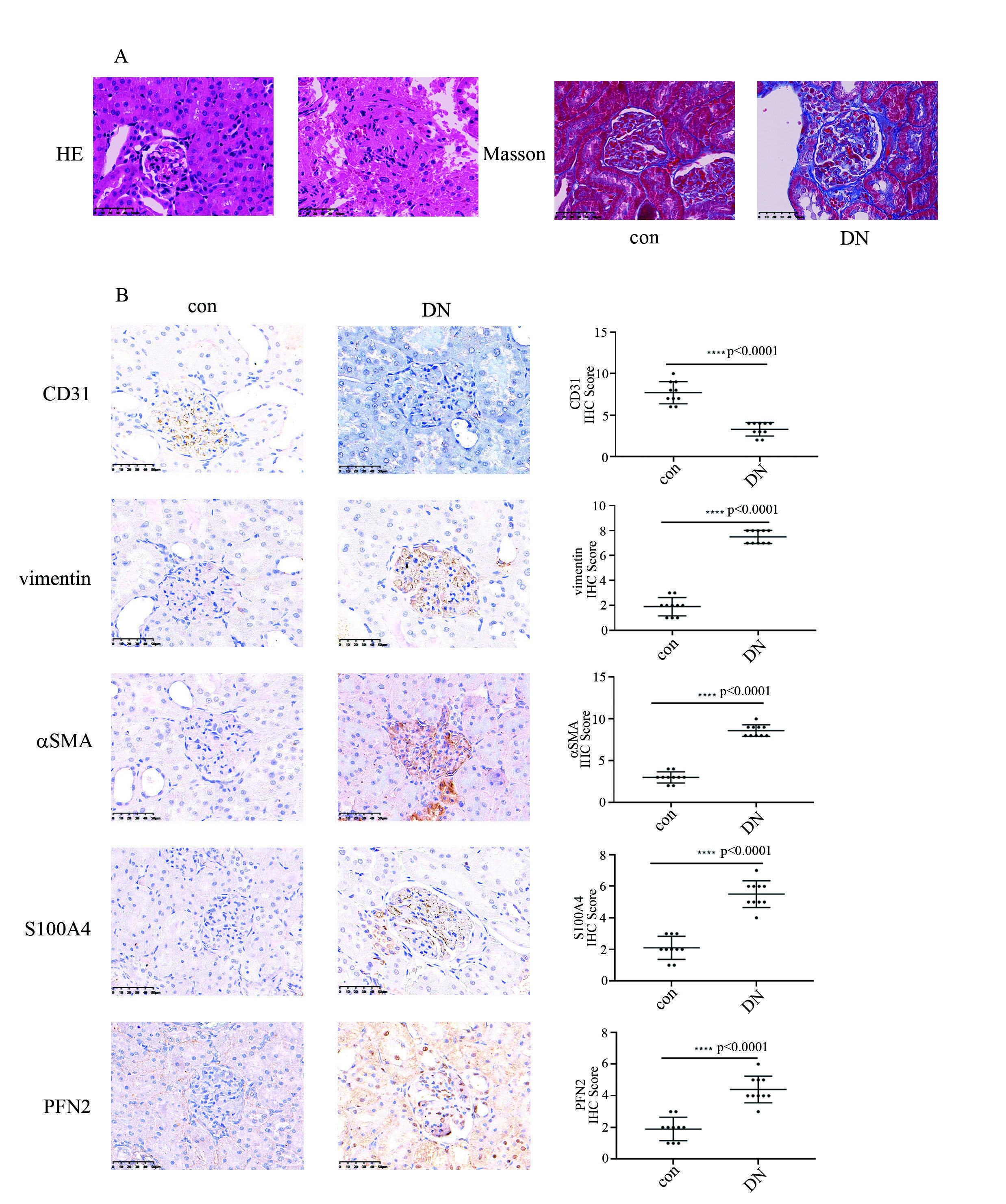

Supplement: Supplementary file 2 — Additional file 2: Figure S1. Occurrence of EndMT and increase in PFN2expression in the glomeruli of DN rats. (A) HE staining and Masson stainingof renal biopsy specimens from DN rats and control rats. Magnification:40 × . Scale bar: 10 μM. (B) Immunostaining of CD31, vimentin, αSMA,S100A4 and PFN2 in renal biopsy specimens of DN rats and control rats.Magnification: 40 × . Scale bar: 10 μM. (* p < 0.05, ** p < 0.01, *** p < 0.001,**** p < 0.0001, n = 10/group). [file 10020_2021_339_MOESM2_ESM.jpg]

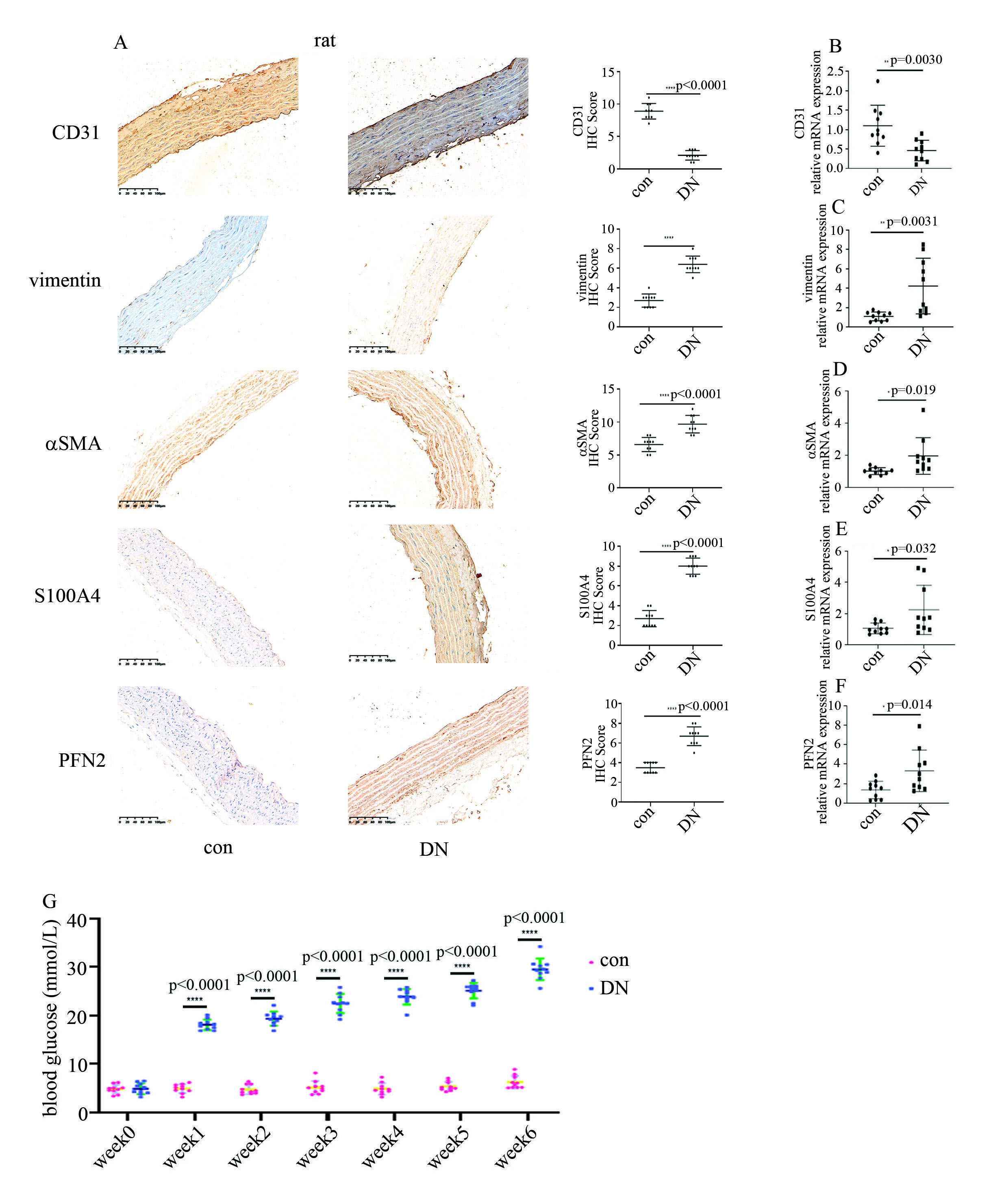

Supplement: Supplementary file 3 — Additional file 3: Figure S2. Occurrence of EndMT and increase in PFN2expression in aortic tissues of DN rats. (A) Immunostaining of CD31,vimentin, αSMA, S100A4 and PFN2 in aortic tissues of DN rats and controlrats. (B) Compared with the control group, the mRNA expression of CD31was decreased in aortic tissues of DN rats. (C) Compared with the controlgroup, the mRNA expression of vimentin was increased in aortic tissuesof DN rats. (D) Compared with the control group, the mRNA expressionof αSMA was increased in aortic tissues of DN rats. (E) Compared with thecontrol group, the mRNA expression of S100A4 was increased in aortictissues of DN rats. (F) Compared with the control group, the mRNA expressionof PFN2 was increased in aortic tissues of DN rats. (G) Blood glucoseevolution in the rats started after the induction of diabetes at 6 weeks.Magnification: 20 × . Scale bar: 20 μM. (* p < 0.05, ** p < 0.01, *** p < 0.001,**** p < 0.0001, n = 10/group). [file 10020_2021_339_MOESM3_ESM.jpg]

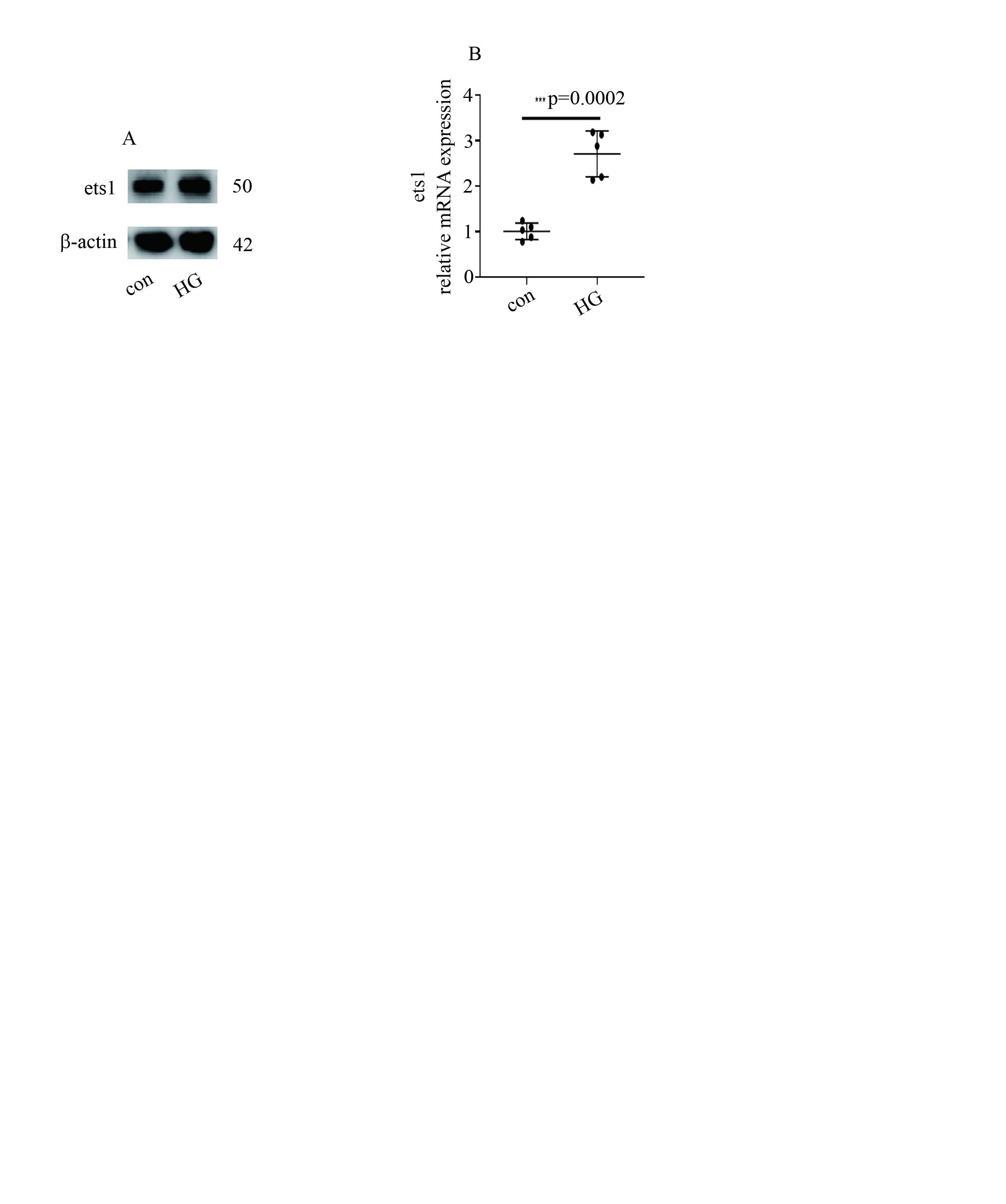

Supplement: Supplementary file 4 — Additional file 4: Figure S3. High glucose upregulated ets1 expressionin the HUVECs. (A) Results from the Western blot analysis of ets1 in theHUVECs with the corresponding treatment. (B) Compared with the controlgroup, the mRNA expression of ets1 was increased in hyperglycemicHUVECs. (* p < 0.05, ** p < 0.01, *** p < 0.001, **** p < 0.0001, n = 5/group). [file 10020_2021_339_MOESM4_ESM.jpg]

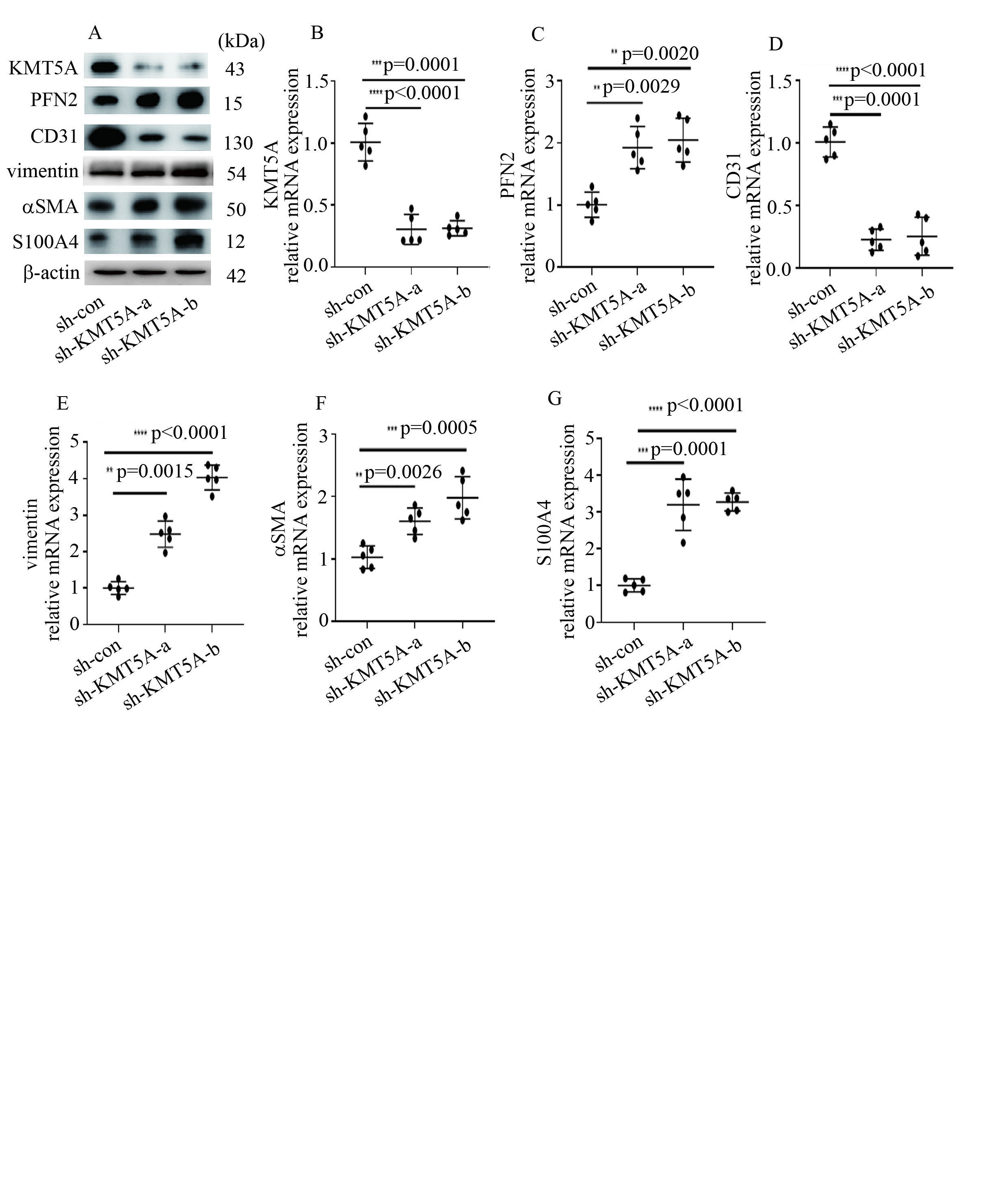

Supplement: Supplementary file 5 — Additional file 5: Figure S4. sh-KMT5A induced EndMT and PFN2 expression inHUVECs. (A) Results from the Western blot analysis of KMT5A, PFN2, CD31,vimentin, αSMA, and S100A4 in the HUVECs with the corresponding treatment.(B) The effects of sh-KMT5A were confirmed by qPCR. (C) Comparedwith the control group, sh-KMT5A increased PFN2 mRNA expression inHUVECs. (D) Compared with the control group, sh-KMT5A decreasedCD31 mRNA expression in HUVECs. (E) Compared with the control group,sh-KMT5A increased vimentin mRNA expression in HUVECs. (F) Comparedwith the control group, sh-KMT5A increased αSMA mRNA expressionin HUVECs. (G) Compared with the control group, sh-KMT5A increasedS100A4 mRNA expression in HUVECs. (* p < 0.05, ** p < 0.01, *** p < 0.001,**** p < 0.0001, n = 5/group). [file 10020_2021_339_MOESM5_ESM.jpg]

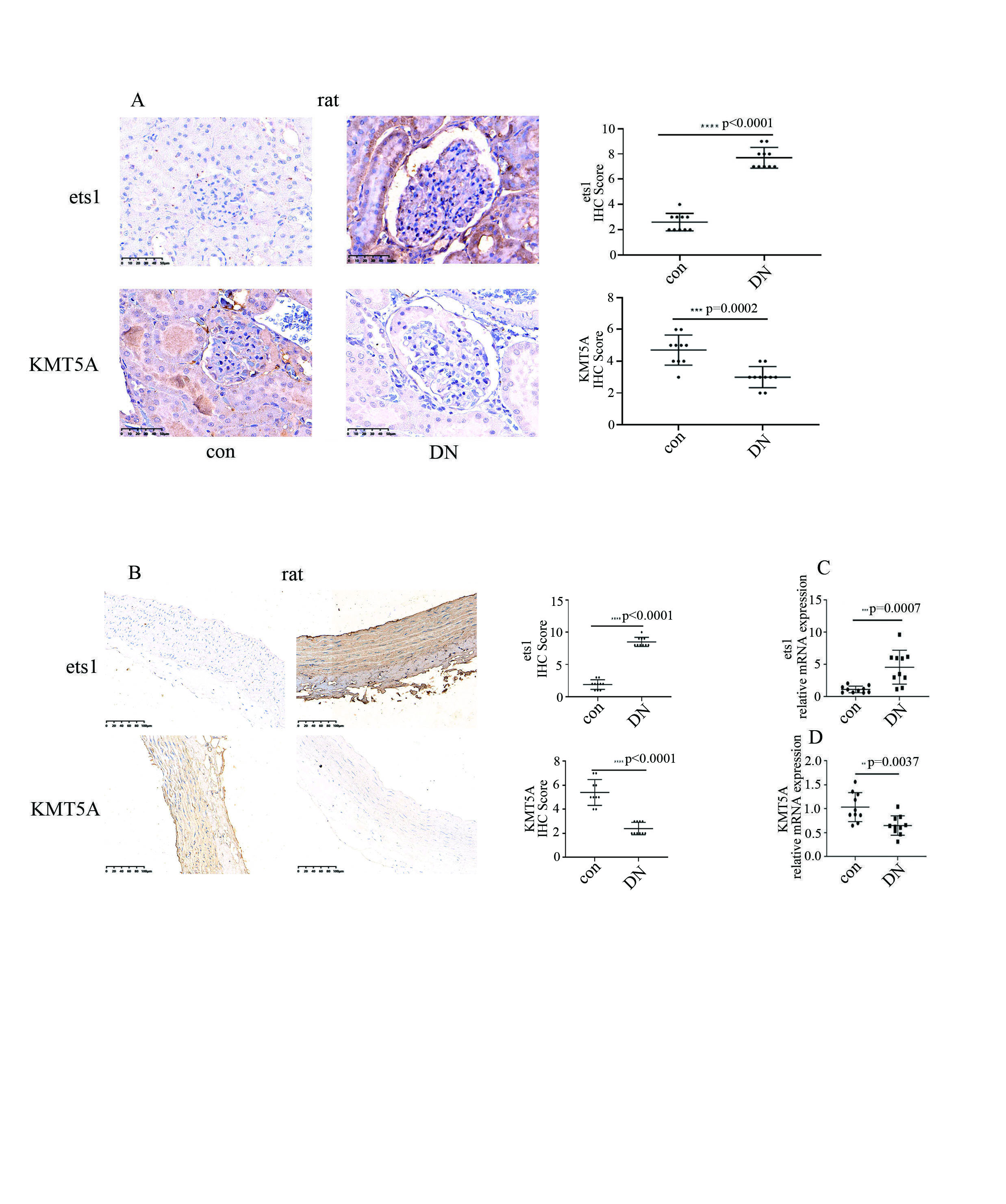

Supplement: Supplementary file 6 — Additional file 6: Figure S5. The expression of ets1 and KMT5A in DN ratsand control rats. (A) Immunostaining of ets1 and KMT5A in renal biopsyspecimens of DN rats and control rats. (Magnification: 40 × . Scale bar:10 μM) (B) Immunostaining of ets1 and KMT5A in aortic tissues of DN ratsand control rats. (Magnification: 20 × . Scale bar: 20 μM) (C) Comparedwith the control group, the mRNA expression of ets1 was increased inaortic tissues of DN rats. (D) Compared with the control group, the mRNAexpression of KMT5A was decreased in aortic tissues of DN rats. (* p < 0.05,** p < 0.01, *** p < 0.001, **** p < 0.0001, n = 10/group). [file 10020_2021_339_MOESM6_ESM.jpg]
